# *In vitro* and *in vivo* Metabolism of a Potent Inhibitor of Soluble Epoxide Hydrolase, 1-(1-Propionylpiperidin-4-yl)-3-(4-(trifluoromethoxy)phenyl)urea

**DOI:** 10.3389/fphar.2019.00464

**Published:** 2019-05-08

**Authors:** Debin Wan, Jun Yang, Cindy B. McReynolds, Bogdan Barnych, Karen M. Wagner, Christophe Morisseau, Sung Hee Hwang, Jia Sun, René Blöcher, Bruce D. Hammock

**Affiliations:** ^1^Department of Entomology and Nematology and UC Davis Comprehensive Cancer Center, University of California, Davis, Davis, CA, United States; ^2^State Forestry Administration Key Open Laboratory, International Center for Bamboo and Rattan, Beijing, China

**Keywords:** soluble epoxide hydrolase inhibitor, TPPU, drug metabolism, *in vitro*, *in vivo*, LC-MS, precursor ion scan, sEH potency

## Abstract

1-(1-Propionylpiperidin-4-yl)-3-(4-(trifluoromethoxy)phenyl)urea (TPPU) is a potent soluble epoxide hydrolase (sEH) inhibitor that is used extensively in research for modulating inflammation and protecting against hypertension, neuropathic pain, and neurodegeneration. Despite its wide use in various animal disease models, the metabolism of TPPU has not been well-studied. A broader understanding of its metabolism is critical for determining contributions of metabolites to the overall safety and effectiveness of TPPU. Herein, we describe the identification of TPPU metabolites using LC-MS/MS strategies. Four metabolites of TPPU (M1–M4) were identified from rat urine by a sensitive and specific LC-MS/MS method with double precursor ion scans. Their structures were further supported by LC-MS/MS comparison with synthesized standards. Metabolites M1 and M2 were formed from hydroxylation on a propionyl group of TPPU; M3 was formed by amide hydrolysis of the 1-propionylpiperdinyl group on TPPU; and M4 was formed by further oxidation of the hydroxylated metabolite M2. Interestingly, the predicted α-keto amide metabolite and 4-(trifluoromethoxy)aniline (metabolite from urea cleavage) were not detected by the LC-MRM-MS method. This indicates that if formed, the two potential metabolites represent <0.01% of TPPU metabolism. Species differences in the formation of these four identified metabolites was assessed using liver S9 fractions from dog, monkey, rat, mouse, and human. M1, M2, and M3 were generated in liver S9 fractions from all species, and higher amounts of M3 were generated in monkey S9 fractions compared to other species. In addition, rat and human S9 metabolism showed the highest species similarity based on the quantities of each metabolite. The presence of all four metabolites were confirmed *in vivo* in rats over 72-h post single oral dose of TPPU. Urine and feces were major routes for TPPU excretion. M1, M4 and parent drug were detected as major substances, and M2 and M3 were minor substances. In blood, M1 accounted for ~9.6% of the total TPPU-related exposure, while metabolites M2, M3, and M4 accounted for <0.4%. All four metabolites were potent inhibitors of human sEH but were less potent than the parent TPPU. In conclusion, TPPU is metabolized via oxidation and amide hydrolysis without apparent breakdown of the urea. The aniline metabolites were not observed either *in vitro* or *in vivo*. Our findings increase the confidence in the ability to translate preclinical PK of TPPU in rats to humans and facilitates the potential clinical development of TPPU and other sEH inhibitors.

## Introduction

The soluble epoxide hydrolase (sEH) is responsible for mediating the metabolism of epoxy fatty acids (EpFAs), such as epoxyeicosatrienoic acids (EETs) (Newman et al., 2005; Decker et al., 2009) and is broadly distributed throughout the mammalian body (Enayetallah et al., 2004). sEH hydrolyzes biologically active EpFAs to their less active corresponding vicinal dihydroxy fatty acids. Cytochrome P450-produced EETs from oxidation of arachidonic acid and other EpFAs are important endogenous anti-inflammatory lipid mediators that resolve inflammation in part via modulating NF-κB signaling (Xu et al., 2006). However, sEH rapidly coverts EETs and other EpFAs to their less active dihydroxy-eicosatrienoic acids (DHETs) *in vivo* (Morisseau and Hammock, 2013). Therefore, a series of potent sEH inhibitors containing a *N,N*′-disubstituted urea have been developed to stabilize the EpFA and increase their residence time to exert their beneficial effects.

Recent studies have yielded a better understanding of the role of sEH in regulating the progression of several diseases in preclinical studies (Luria et al., 2011; Imig, 2012, 2015; Wang et al., 2013, 2018; Ren et al., 2018). The inhibition of sEH demonstrates a promising approach to treat various diseases, including hypertension (Imig et al., 2005; Anandan et al., 2011), inflammation (Davis et al., 2011; Bastan et al., 2018), diabetes (Lorthioir et al., 2012; Hu et al., 2017), neuropathic pain (Hammock et al., 2011; McReynolds et al., 2016; Wagner K. et al., 2017; Wagner K. M. et al., 2017), and central nervous system (CNS) disorders (Simpkins et al., 2009; Ren et al., 2016; Zarriello et al., 2018). Growing evidence suggests that EpFAs reduce endoplasmic reticulum stress as a common underlying mechanism for these therapeutic effects (Yu et al., 2015; Inceoglu et al., 2017). In fact, several selective sEH inhibitors such as AR9281 and GSK2256294 have reached clinical trials (Chen et al., 2012; Lazaar et al., 2016). These Phase I clinical trials did not reveal any toxicity limitations for those novel drug candidates to be eventually clinically-approved.

Among the sEH inhibitors optimized for use *in vivo*, 1-(4-trifluoro-methoxy-phenyl)-3-(1-propionylpiperidin-4-yl) urea (TPPU) is widely used because of its good potency, pharmacokinetics, and biological activity (Rose et al., 2010; Ulu et al., 2012; Liu et al., 2013) without apparent non-specific binding (Lee et al., 2014). It tightly binds to the recombinant human sEH with a low nanomolar K_i_ (0.9 ± 0.1 nM) and a slow k_off_ (10.5 × 10^−4^ s^−1^) indicating its high inhibition potency and target occupancy against human sEH (Lee et al., 2014). Therefore, the pharmacological effects of TPPU have been studied extensively in a number of animal models (Qiu et al., 2011; Shen and Hammock, 2012; Bettaieb et al., 2015; Harris et al., 2015; Goswami et al., 2016; Hashimoto, 2016; Supp et al., 2016; Zhou et al., 2016, 2017; Chen et al., 2017; Wu et al., 2017; Huang et al., 2018; Napimoga et al., 2018; Tu et al., 2018). Notably, TPPU treatment can significantly decrease infarct volume, reduce neurologic deficits and improve sensorimotor function in transient middle cerebral artery occlusion in rats (Tu et al., 2018); reduce 1-methyl-4-phenyl-1,2,3,6-tetrahydropyridine (MPTP) induced neurotoxicity in the mouse striatum (Huang et al., 2018; Ren et al., 2018); and act as a rapid antidepressant in murine depression models (Hashimoto, 2016; Wu et al., 2017). It is also worth mentioning that TPPU both acts synergistically with and reduces the side effects of non-steroidal anti-inflammatory drugs (NSAIDs) (Qiu et al., 2011; Shen and Hammock, 2012). For instance, the severity of gastrointestinal ulcers induced by diclofenac can be prevented by TPPU (Goswami et al., 2016).

Despite its wide use in various animal disease models, the metabolism of TPPU has not been well-studied. The pharmacokinetic profile of TPPU has been investigated in several different species, and it shows high blood levels are achieved *in vivo* and are associated with long half-life of the parent compound (Tsai et al., 2010; Ulu et al., 2012; Liu et al., 2013; Lee et al., 2014). As more studies uncover the potential therapeutic benefits of sEH inhibition, it becomes essential to characterize the metabolism of this sEH inhibitor for determining appropriate dose of TPPU and the contributions of its metabolites to its overall safety and effectiveness. Therefore, we investigated the *in vitro* and *in vivo* metabolism of TPPU.

## Materials and Methods

### Animals and Chemicals

Animal experiments were approved by the Animal Use and Care Committee of University of California, Davis. Male rats (Sprague Dawley, 250–300 g) were purchased from the Charles River Laboratories (CA). Liver S9 fractions from human, monkey, dog, rat, and mouse were purchased from XenoTech, LLC (Lenexa, KS). TPPU and its four metabolites as well as 1-(1-acetylpiperidin-4-yl)-3-(4-(trifluoromethyl)phenyl)urea (TAPU) and 12-(3-cyclohexyl-ureido)-dodecanoic acid (CUDA) were synthesized in house and their syntheses are described in the supplementary material. Reagents for the NADPH regeneration system, including beta-nicotinamide adenine dinucleotide phosphate sodium salt (NADP^+^), D-glucose-6-phosphate dehydrogenase (G6PDH), D-glucose-6-phosphate monosodium salt (G6P), anhydrous magnesium chloride (MgCl_2_), sodium chloride (NaCl), ethylenediaminetetraacetic acid (EDTA), and glacial acetic acids were obtained from Sigma (St. Louis, MO). LC-MS grade water, methanol (MeOH), acetonitrile, ethyl acetate (EA), reagent grade monobasic monohydrate sodium phosphate, and anhydrous dibasic sodium phosphate were purchased from Fisher Scientific (Pittsburgh, PA).

### Synthesis of TPPU Metabolites, Scheme 1

The synthesis started with preparation of the common intermediate **compound 1 (I1)** via conventional triphosgene mediated unsymmetrical urea (**M3**) formation between 4-(trifluoromethoxy)aniline and *tert*-butyl 4-aminopiperidine-1-carboxylate followed by deprotection with TFA. **M1** was synthesized in 4.5% yield by treatment of the intermediate **I1** with 2-hydroxypropanoic acid and PyBOP in the presence of triethylamine. Acylation of the intermediate **I1** with oxetan-2-one produced metabolite **M2**. Acylation of **I1** with ethyl malonyl chloride followed by hydrolysis of the ester function provided the last metabolite **M4**. Acylation of **I1** with pyruvic acid gave **α-keto amide**. Starting materials were purchased from one of the following commercial sources: Sigma Aldrich Chemical Co. (Milwaukee, WI), Fisher Scientific (Houston, TX), Eanmine LLC (Monmouth Jct, NJ), Oakwood Chemical (Estill, SC). All reactions were carried out under an atmosphere of dry nitrogen. All chemicals purchased from commercial sources were used as received without further purification. Analytical thin layer chromatography (TLC) was performed on Merck TLC silica gel 60 F254 plates. Flash chromatography was performed on silica gel (230–400 mesh) from Macherey Nagel. NMR spectra were recorded on Varian VNMRS 600, Inova 400, or Bruker Avance III 800 MHz instruments. Multiplicity is described by the abbreviations b = broad, s = singlet, d = doublet, t = triplet, q = quartet, p = pentet, m = multiplet. Chemical shifts are given in ppm. ^1^H NMR spectra are referenced to the residual solvent peak at δ = 7.26 (CDCl_3_) or 3.31 (CD_3_OD). ^13^C NMR spectra are referenced to the solvent peak at δ = 77.16 (CDCl_3_) or 49.00 (CD_3_OD). HRMS spectra were recorded and are presented on Thermo Electron LTQ-Orbitrap XL Hybrid MS in ESI. All synthesized compounds were >95% pure based on NMR and LC-MS data.

**Scheme 1:**
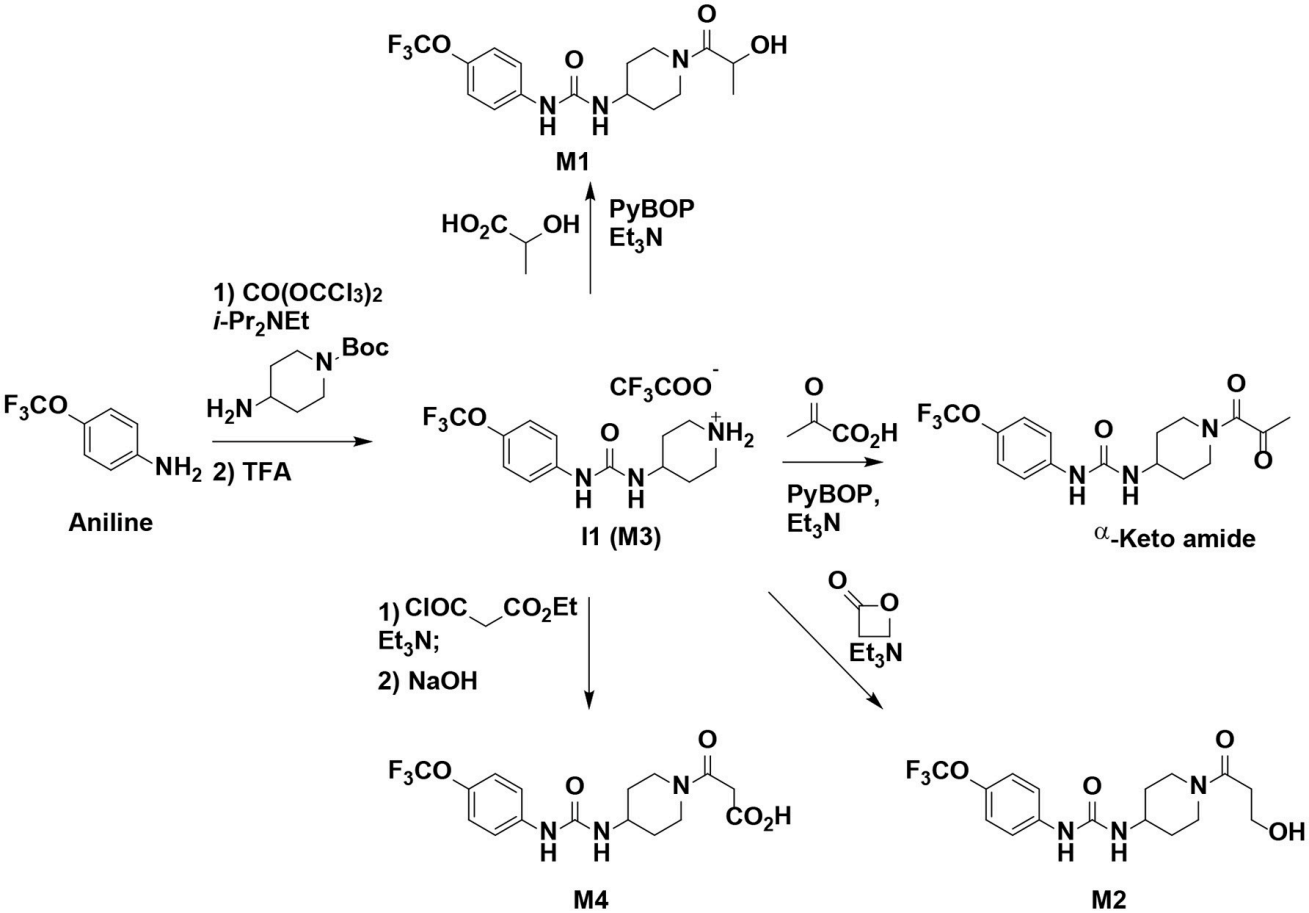
Synthesis of standard metabolites of TPPU.

#### *tert*-butyl 4-(3-(4-(trifluoromethoxy)phenyl)ureido) piperidine-1-carboxylate

A solution of 4-(trifluoromethoxy)aniline (1.77 g, 10 mmol, 1 equiv) and diisopropylethyl amine (1.419 g, 11 mmol, 1.1 equiv) in CH_2_Cl_2_ (15 mL) was added dropwise to the solution of triphosgene (1.13 g, 3.8 mmol, 0.38 equiv) in CH_2_Cl_2_ (20 mL) at 0°C. The reaction mixture was stirred for 40 min and then the solution of *tert*-butyl 4-aminopiperidine-1-carboxylate (2 g, 10 mmol, 1 equiv) and diisopropylethyl amine (2.58 g, 20 mmol, 2 equiv) in CH_2_Cl_2_ (10 mL) was added dropwise. The reaction mixture was stirred overnight, quenched with water/brine (1:1, 30 mL), and few drops of 3 M HCl (until pH ~ 3), and extracted with CH_2_Cl_2_ (3 × 30 mL). Combined extracts were dried over MgSO_4_, filtered, and evaporated under reduced pressure. Purification of the residue by flash column chromatography (EtOAc/hexanes = 1:1 → EtOAc) gave pure product as a pale-tan solid (3.22 g, 80 %). ^1^H NMR (600 MHz, CDCl_3_) δ 7.36 (bs, 1H), 7.33 (d, *J* = 9.0 Hz, 2H), 7.11 (d, *J* = 8.7 Hz, 2H), 5.30 (bs, 1H), 3.96 (bs, 2H), 3.79 (m, 1H), 2.85 (t, *J* = 12.6 Hz, 2H), 1.85 (m, 3H), 1.45 (s, 9H), 1.22 (bs, 2H). ^13^C NMR (151 MHz, CDCl_3_) δ 155.2, 155.0, 144.4, 137.9, 123.2, 122.0, 121.5, 120.4, 119.8, 118.1, 80.4, 47.1, 42.8, 32.8, 28.6.

#### 4-(3-(4-(trifluoromethoxy)phenyl)ureido)piperidin-1-ium 2,2,2-trifluoroacetate I1

*tert*-butyl 4-(3-(4-(trifluoromethoxy)phenyl)ureido)piperidine-1-carboxylate (3.2 g, 7.94 mmol, 1 equiv) was dissolved in the mixture of CH_2_Cl_2_ (5 mL) and TFA (5 mL) and the resulting mixture was stirred at room temperature for ~3 h and evaporated. The resulting product was used in the next steps without purification and had amine:TFA ratio of ~1:4.48.

#### 1-(1-(2-hydroxypropanoyl)piperidin-4-yl)-3-(4-(trifluoromethoxy)phenyl)urea M1

A mixture of 4-(3-(4-(trifluoromethoxy)phenyl)ureido)piperidin-1-ium 2,2,2-trifluoroacetate **I1** (423 mg, 0.55 mmol, 1 equiv), 2-hydroxypropanoic acid (76 mg, 0.72 mmol, 1.3 equiv), PyBOP (374 mg, 0.72 mmol, 1.3 equiv), and Et_3_N (391 mg, 3.87 mmol, 7 equiv) was stirred at room temperature for 16 h, quenched with water and extracted with EtOAc (3 × 15 mL). Combined extracts were dried over MgSO_4_, filtered, and evaporated under reduced pressure. Purification of the residue by flash column chromatography (EtOAc) gave pure product as a pale-tan solid (9.3 mg, 4.5 %). ^1^H NMR (600 MHz, CD_3_OD) δ 7.44 (d, *J* = 8.9 Hz, 2H), 7.15 (d, *J* = 8.7 Hz, 2H), 4.60 (q, *J* = 6.6 Hz, 1H), 4.47–4.28 (m, 1H), 3.95 (m, 1H), 3.84 (m, 1H), 3.49–3.34 (m, 1H), 3.22 (m, 1H), 3.03–2.84 (m, 1H), 2.06–1.92 (m, 2H), 1.49–1.27 (m, 5H). ^13^C NMR (151 MHz, CD_3_OD) δ 175.93, 175.92, 174.84, 174.62, 174.57, 171.06, 171.04, 157.08, 144.98, 140.04, 124.46, 122.77, 122.62, 121.08, 120.91, 119.40, 70.17, 68.84, 67.66, 67.63, 67.01, 65.82, 65.73, 48.07, 47.98, 47.30, 47.26, 45.05, 44.77, 42.34, 42.15, 33.91, 33.69, 33.10, 32.96, 27.00, 26.95, 24.84, 24.79, 21.11, 20.84, 20.48, 20.14. HRMS (ESI), calculated for C_16_H_21_F_3_N_3_O_4_ ([M+H]^+^) *m/z* 376.1479, found *m/z* 376.1481.

#### 1-(1-(3-hydroxypropanoyl)piperidin-4-yl)-3-(4-(trifluoromethoxy)phenyl)urea M2

A mixture of 4-(3-(4-(trifluoromethoxy)phenyl)ureido)piperidin-1-ium 2,2,2-trifluoroacetate **I1** (400 mg, 0.52 mmol, 1 equiv), oxetan-2-one (150 mg, 2.09 mmol, 2 equiv), Et_3_N (634 mg, 6.27 mmol, 12 equiv) and CH_2_Cl_2_ (1 mL) was stirred for 4 days and directly chromatographed (EtOAc → EtOAc:MeOH = 99:1 → 98:2) to give pure product as a pail-tan solid (21.9 mg, 11 %). ^1^H NMR (600 MHz, CD_3_OD) δ 7.44 (d, *J* = 9.1 Hz, 2H), 7.15 (d, *J* = 8.9 Hz, 2H), 4.41 (d, *J* = 13.7 Hz, 1H), 3.96 (d, *J* = 13.9 Hz, 1H), 3.83 (t, *J* = 6.1 Hz, 2H), 3.82 (m, 1H), 3.25 (ddd, *J* = 14.1, 11.4, 2.9 Hz, 1H), 2.91 (t, *J* = 11.4 Hz, 1H), 2.64 (m, 2H), 2.01 (m, 1H), 1.95 (m, 1H), 1.46 (m, 1H), 1.38 (m, 1H). ^13^C NMR (151 MHz, CD_3_OD) δ 173.0, 157.2, 145.0, 140.2, 122.8, 122.7, 120.9, 59.3, 48.1, 45.8, 41.8, 33.9, 33.1. HRMS (ESI), calculated for C_16_H_20_F_3_N_3_NaO_4_ ([M+Na]^+^) *m/z* 398.1298, found *m/z* 398.1300.

#### Ethyl 3-oxo-3-(4-(3-(4-(trifluoromethoxy)phenyl) ureido)piperidin-1-yl)propanoate

A solution of ethyl malonyl chloride (89 mg, 0.59 mmol, 1.1 equiv) in CH_2_Cl_2_ (0.2 mL) was added dropwise to a cooled to 0°C solution of 4-(3-(4-(trifluoromethoxy)phenyl)ureido)piperidin-1-ium 2,2,2-trifluoroacetate **I1** (412 mg, 0.54 mmol, 1 equiv) and Et_3_N (326 mg, 3.23 mmol, 6 equiv) in CH_2_Cl_2_ (3 mL). The reaction mixture was stirred at room temperature for 3 h and directly chromatographed (EtOAc → EtOAc:MeOH = 99:1 → 98:2) to give pure product as a pail-tan solid (144 mg, 64%). ^1^H NMR (600 MHz, CDCl_3_) δ 7.88 (s, 1H), 7.37 (d, *J* = 9.0 Hz, 2H), 7.07 (d, *J* = 8.7 Hz, 2H), 5.69 (d, *J* = 7.6 Hz, 1H), 4.38 (d, *J* = 13.2 Hz, 1H), 4.14 (q, *J* = 7.1 Hz, 2H), 3.89 (m, 1H), 3.64 (d, *J* = 14.7 Hz, 1H), 3.50 (d, *J* = 15.7 Hz, 1H), 3.43 (d, *J* = 15.7 Hz, 1H), 3.16 (ddd, *J* = 14.1, 11.4, 2.8 Hz, 1H), 2.85 (ddd, *J* = 14.0, 11.5, 3.0 Hz, 1H), 2.03 (m, 1H), 1.92 (m, 1H), 1.39–1.23 (m, 2H), 1.22 (t, *J* = 7.2 Hz, 3H). ^13^C NMR (151 MHz, CDCl_3_) δ 168.0, 165.2, 155.3, 144.0, 138.4, 123.1, 121.8, 121.4, 119.9, 119.7, 118.0, 61.9, 46.5, 45.5, 41.3, 41.2, 33.0, 31.9, 14.1.

#### 3-oxo-3-(4-(3-(4-(trifluoromethoxy)phenyl)ureido) piperidin-1-yl)propanoic acid M4

A solution of ethyl 3-oxo-3-(4-(3-(4-(trifluoro methoxy)phenyl)ureido)piperidin-1-yl)propanoate (144 mg, 0.345 mmol, 1 equiv) and NaOH (10 M, 0.345 mL, 3.45 mmol, 10 equiv) in MeOH was stirred at room temperature for 20 h and evaporated. The residue was dissolved in water and acidified to pH ~ 3. The precipitate was filtered and dried under vacuum to give pure product as white solid (105.8 mg, 79%). ^1^H NMR (600 MHz, CD_3_OD) δ 7.44 (d, *J* = 9.0 Hz, 2H), 7.15 (d, *J* = 8.6 Hz, 2H), 4.37 (d, *J* = 13.3 Hz, 1H), 3.83 (m, 2H), 3.58 (d, *J* = 16.3 Hz, 1H), 3.51 (d, *J* = 16.2 Hz, 1H), 3.26 (ddd, *J* = 14.2, 11.3, 3.0 Hz, 1H), 2.94 (ddd, *J* = 13.9, 11.4, 3.1 Hz, 1H), 1.98 (m, 2H), 1.51 (m, 1H), 1.40 (m, 1H). ^13^C NMR (151 MHz, CD_3_OD) δ 171.1, 167.5, 157.1, 145.0, 140.0, 122.8, 122.7, 121.1, 120.9, 47.9, 46.4, 41.9, 41.2, 33.5, 32.8. HRMS (ESI), calculated for C_16_H_17_F_3_N_3_O_5_ ([M-H]^−^) *m/z* 388.1126, found *m/z* 388.1094.

#### 1-(1-(2-oxopropanoyl)piperidin-4-yl)-3-(4-(trifluoromethoxy)phenyl)urea α-keto amide

A mixture of 4-(3-(4-(trifluoromethoxy)phenyl)ureido)piperidin-1-ium 2,2,2-trifluoroacetate **I1** (347 mg, 0.45 mmol, 1 equiv), puryvic acid (52 mg, 0.59 mmol, 1.3 equiv), PyBOP (307 mg, 0.59 mmol, 1.3 equiv), and Et_3_N (321 mg, 3.18 mmol, 7 equiv) was stirred at room temperature for 20 h, and directly chromatographed (EtOAc → EtOAc:MeOH = 99:1 → 98:2) to give pure product as a pail-tan solid (71.2 mg, 42%). ^1^H NMR (600 MHz, CD_3_OD) δ 7.43 (d, *J* = 9.0 Hz, 1H), 7.15 (d, *J* = 8.8 Hz, 1H), 4.28 (d, *J* = 13.5 Hz, 1H), 3.86 (tt, *J* = 10.5, 4.1 Hz, 1H), 3.72 (d, *J* = 14.0 Hz, 1H), 3.24 (ddt, *J* = 14.1, 11.3, 2.3 Hz, 1H), 3.04–2.96 (m, 1H), 2.39 (s, 2H), 2.06–1.94 (m, 1H), 1.54–1.38 (m, 1H). ^13^C NMR (151 MHz, CD_3_OD) δ 200.2, 167.4, 157.0, 145.0, 140.0, 124.5, 122.8, 122.6, 121.1, 120.9, 119.4, 47.8, 45.7, 41.3, 33.7, 32.8, 27.6. HRMS (ESI), calculated for C_16_H_18_F_3_N_3_NaO_4_ ([M+Na]^+^) *m/z* 396.1142, found *m/z* 396.1160.

### *In vitro* Study

The *in vitro* study of TPPU metabolism was conducted following previous methods as described (Watanabe and Hammock, 2001). Briefly, 1 μL of TPPU (1 mM in DMSO; [TPPU]_final_ = 10 μM) was mixed with 94 μL of diluted liver S9 fractions (final, 0.05 mg protein/mL) from human, monkey, dog, rat, and mouse and incubations were conducted in a 10 mL glass vial. After 5 min preincubation at 37°C, the reaction was initiated by adding 5 μL of NADPH regeneration solution. The NADPH regeneration solution was prepared by mixing 50 μL of NADP^+^ (100 mM), 50 μL of G6P (500 mM), 50 μL of G6PDH (100 Unit/mL), and 100 μL of sodium phosphate buffer. Incubation without NADPH regeneration solution was used as control. Reactions were kept for 2 h and terminated by addition of 100 μL of ice-cold methanol containing CUDA (200 nM) as an internal standard. The mixture was followed by vigorous mixing for 2 min and centrifuging at 10,000^*^g for 10 min. Each supernatant was transferred to an HPLC vial and kept under 4°C until LC-MS analysis.

### *In vivo* Study

Healthy male rats (Sprague Dawley, *n* = 4) were housed in temperature-controlled housing with *ad libitum* standard chow and drinking water. Each rat received TPPU (dissolved in 0.5 mL PEG400) at a dose of 10 mg/kg by oral gavage in order to generate significant amounts of metabolites while corresponding to previous efficacious concentration doses. Both blood and urine samples were collected. Blood samples (10 μL) at time points of 0 (before dosing), 0.25, 0.5, 1, 2, 4, 8, 12, 24, 48, and 72 h post dose were collected from the tail vein. Collected blood samples were transferred to Eppendorf tubes containing 90 μL of EDTA solution (0.1% EDTA and 0.1% acetic acid) and were immediately shaken to avoid blood coagulation. The urine and feces samples were passively collected at the time points of 8, 12, 24, 48, and 72 h post dose. TAPU (10 μL) of 1 μM in methanol was added in each blood and urine sample as an internal standard solution. The blood sample extraction was performed with 200 μL ethyl acetate by liquid-liquid extraction. The representative urine samples were subjected to protein precipitation prior to analysis of TPPU and Phase I metabolites. Briefly, urine (10 μL) was treated with 50 μL acetonitrile. After vortex mixing and centrifugation, the supernatant was collected. The feces samples (50–100 mg) were extracted using 600 μL extraction solution (MeOH: EA = 1:1). The mixtures were kept at −20°C overnight to give efficient extraction of TPPU and its metabolites. On day 2, after vortex mixing and centrifugation, the supernatant was collected. The remaining residue was extracted an additional time with 300 μL extraction solution, and the supernatant was combined after centrifugation. The collected supernatants were dried using a speed vacuum concentrator and reconstituted in 50 μL of 100 nM CUDA solution in methanol. In addition, urine (10 μL) was diluted with water (50 μL) and then subjected to analysis of glucuronide or sulfate conjugates.

### LC-MS/MS Analysis

LC-MS/MS analysis was conducted on an Agilent SL 1200 series LC system (Agilent, Palo Alto, CA) connected to a 4,000 Qtrap mass spectrometer (Applied Biosystems Instrument Corporation, Foster City, CA) with Turbo V ion source. Liquid chromatography was performed on a Kinetex^®^ C18 column (100 × 2.1 mm, 1.7 μm). Three μL solution was injected onto the column held at 45°C for analysis. Mobile phase A and B were water with 0.1% acetic acid and acetonitrile with 0.1% acetic acid, respectively. The LC flow rate was 300 μL/min. The LC gradient for the *in vivo* study is given in Table S1. For *in vitro* study, non-volatile salts were diverted from the mass spectrometer using condition listed in Table S2.

The mass spectrometer was operated in both negative and positive modes. Ion source parameters were optimized and are listed in Table S3. Three different scan types were employed to perform mass spectrometry analysis, including product ion scan, precursor ion scan (optimized parameters in Table S4) and MRM scan (optimized parameters in Table S5).

### sEH Inhibition Potency of TPPU and Its Metabolites

The IC_50_ is the concentration of a compound that reduces the sEH activity by 50%. This was measured here using cyano(6-methoxynaphthalen-2-yl)methyl ((3-phenyloxiran-2-yl)methyl) carbonate (CMNPC) as substrate (Morisseau et al., 2002). Briefly, recombinant human sEH [1 nM in sodium phosphate buffer (0.1 M, pH = 7.4, 0.1 mg/mL BSA)] was incubated with each analyte (0.1 < [I]_final_ < 10,000 nM) at 30°C for 5 min. Then, the reaction was started with the addition to CMNPC ([S]final = 5 μM). The reaction was monitored kinetically for 10 min at 30°C. The formation of the fluorescent product 6-methoxynaphthaldehyde (λ_excitation_ = 330 nm, λ_emission_ = 465 nm, 30°C) was measured every 30 s. IC_50_ values were determined by regression of the remaining activity in function of the inhibitor concentrations. Assays were run in triplicate and IC_50_ values are the averages of three replicates.

## Results

### Identification of Phase I Metabolites of TPPU by LC-MS/MS

Based on chemical structure and plausible metabolic pathways, we firstly predicted possible metabolites of TPPU (Table 1). There are multiple sites that can be subject to Phase I reactions. For example, oxidation at the carbon center, including both the aliphatic groups and aromatic ring, and oxidation at a nitrogen center can generate hydroxylated TPPU metabolites. M1, M2, and compounds 1–3 are metabolites formed from hydroxylation on aliphatic groups. Compounds 4–5 are potentially formed from hydroxylation on the two nitrogen atoms in the urea. Compounds 6–7 could be formed from hydroxylation on the aromatic ring. These hydroxylated TPPU metabolites can be potentially oxidized further to yield additional TPPU metabolites (M4, α-keto amide, and compounds 8–12). In addition, hydrolysis or oxidation can occur on the amide, and urea groups to form additional metabolites. M3 is formed from the amide hydrolysis of the 1-propionylpiperdine residue. The 4-trifluoro-methoxy-aniline and compound 13 could result from the urea cleavage. The amide hydrolysis of compound 13 can generate compound 14. Finally, it is likely that TPPU and some of its Phase I metabolites could be conjugated to form their respective Phase II metabolites. In this study, we employed LC-MS/MS methods with assistance of synthetic standard metabolites to identify Phase I metabolites of TPPU. Analysis of Phase II metabolism of TPPU is quite complicated and beyond the scope of this study. As a preliminary study of its possible Phase II metabolites, a neutral loss scan of 176 and precursor ion scan of 97 were used to screen glucuronide and sulfonate-conjugated metabolites in rat urine, respectively. TPPU metabolites in rat feces and urine will be further analyzed after enzymatic hydrolysis and described separately.

**Table 1:** Predictive TPPU metabolites.

**Compound ID**	**Structure**	**Available authentic standard**
TPPU	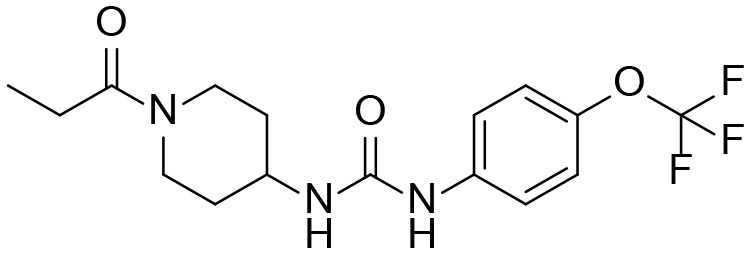	^+^
M1	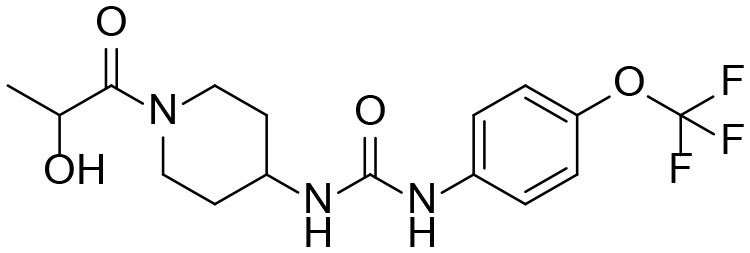	^+^
M2	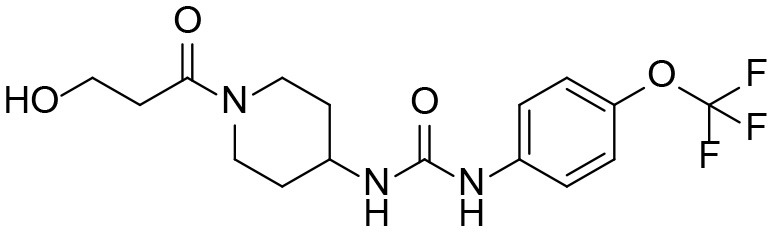	^+^
M3	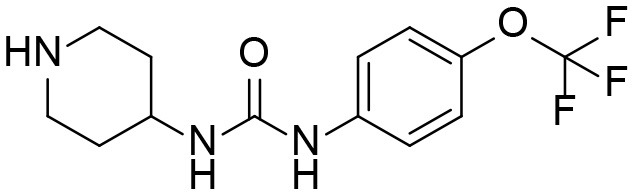	^+^
M4	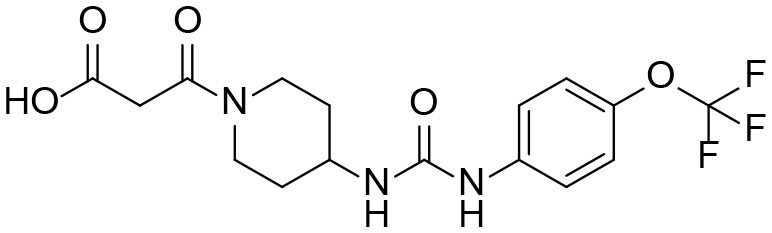	^+^
α-Keto amide	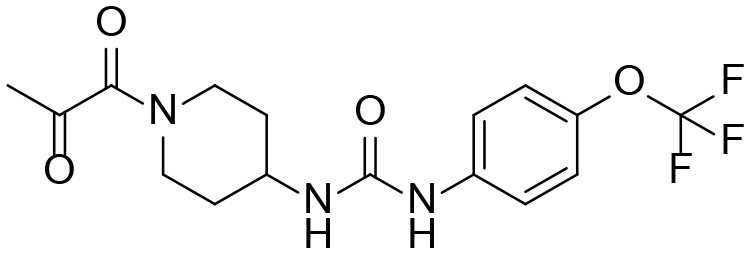	^+^
Aniline	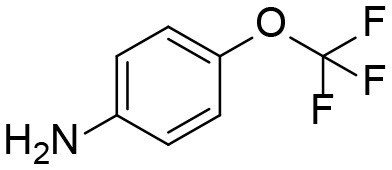	^+^
1	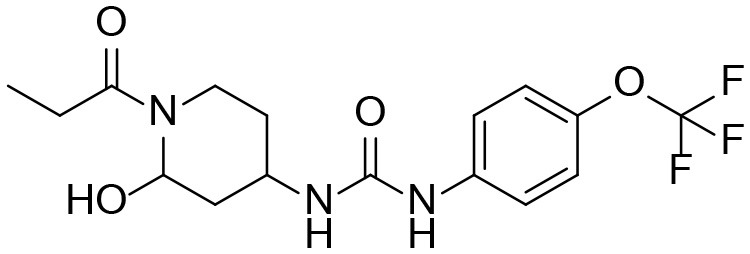	–
2	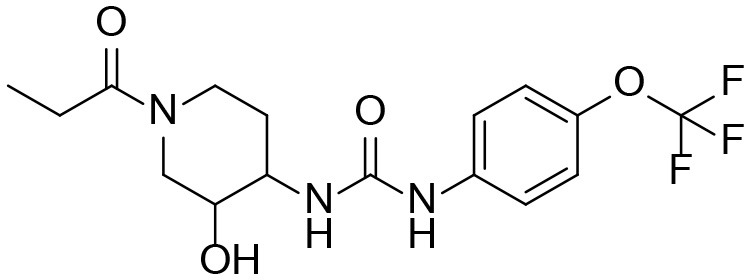	–
3	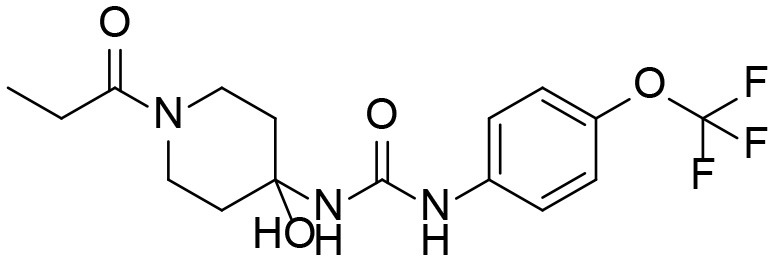	–
4	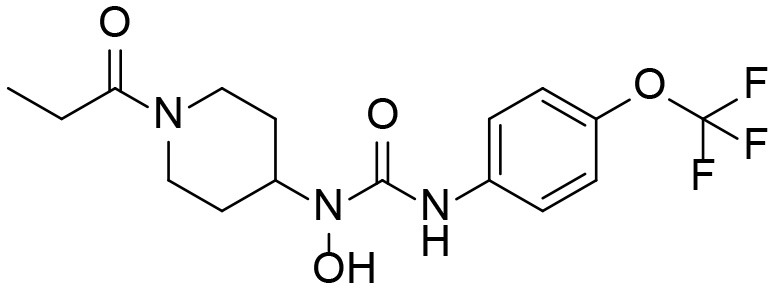	–
5	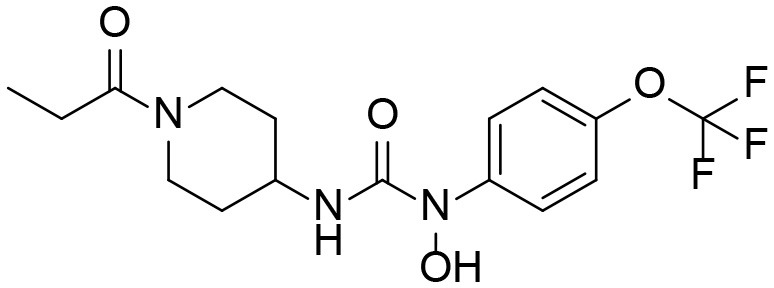	–
6	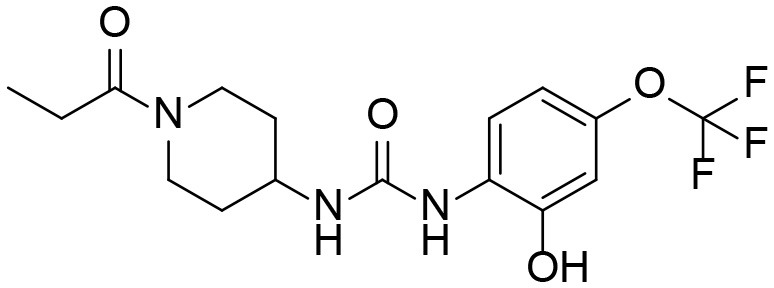	–
7	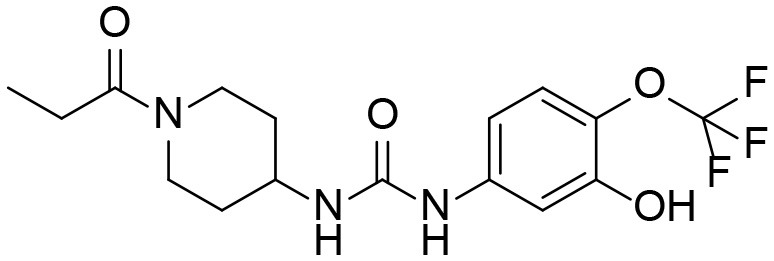	–
8	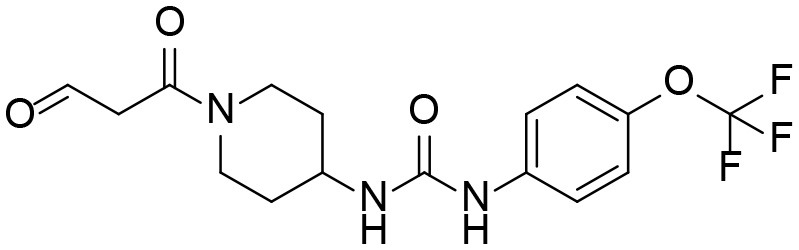	–
9	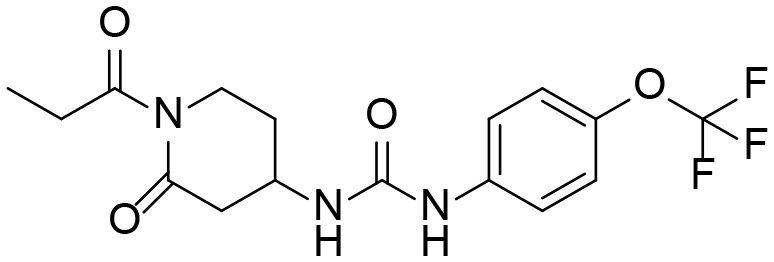	–
10	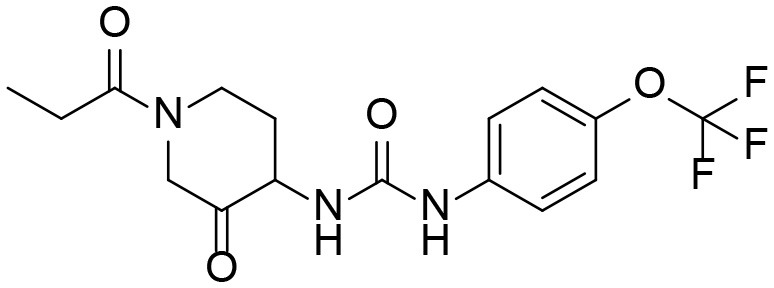	–
11	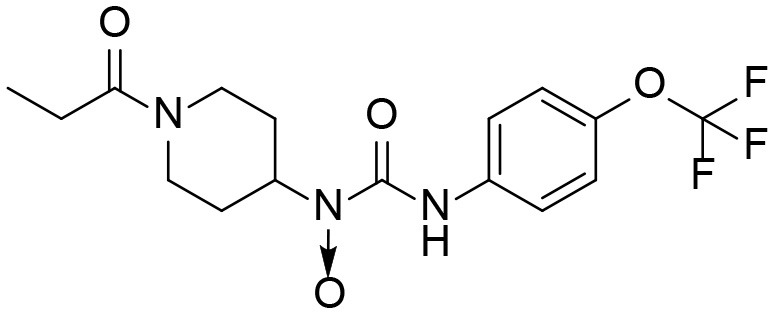	–
12	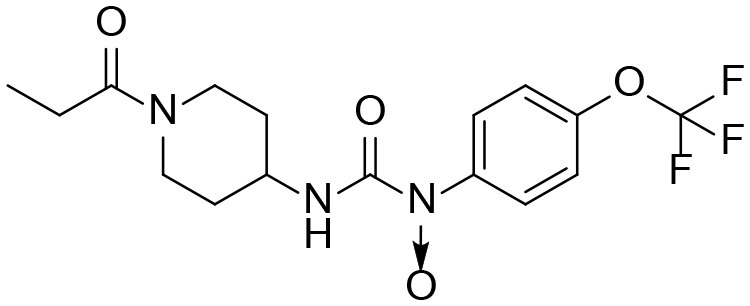	–
13	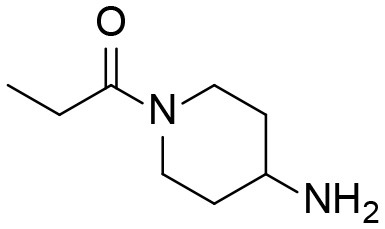	–
14	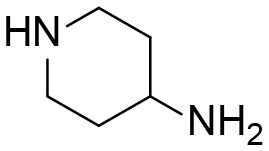	–

*M1–M4 are detectable metabolites. α-Keto amide, aniline, and 1–14 are possible degradation products of TPPU not detected in vitro and in vivo*.

+ *Standard available*.

− *Standard not available*.

#### Analysis of TPPU Metabolites Formed by Oxidation and Amide Hydrolysis

**Figure 1:**
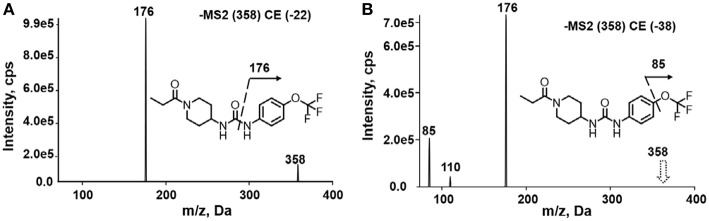
Negative-ion mode ESI tandem mass spectra of TPPU. MS/MS fragmentation was conducted under **(A)** low and **(B)** high collision-induced dissociation (CID) energy. The CID energies were optimized to 22 and 38 eV to obtain highest signals of fragment ions at *m/z* 176 and 85, respectively.

**Figure 2:**
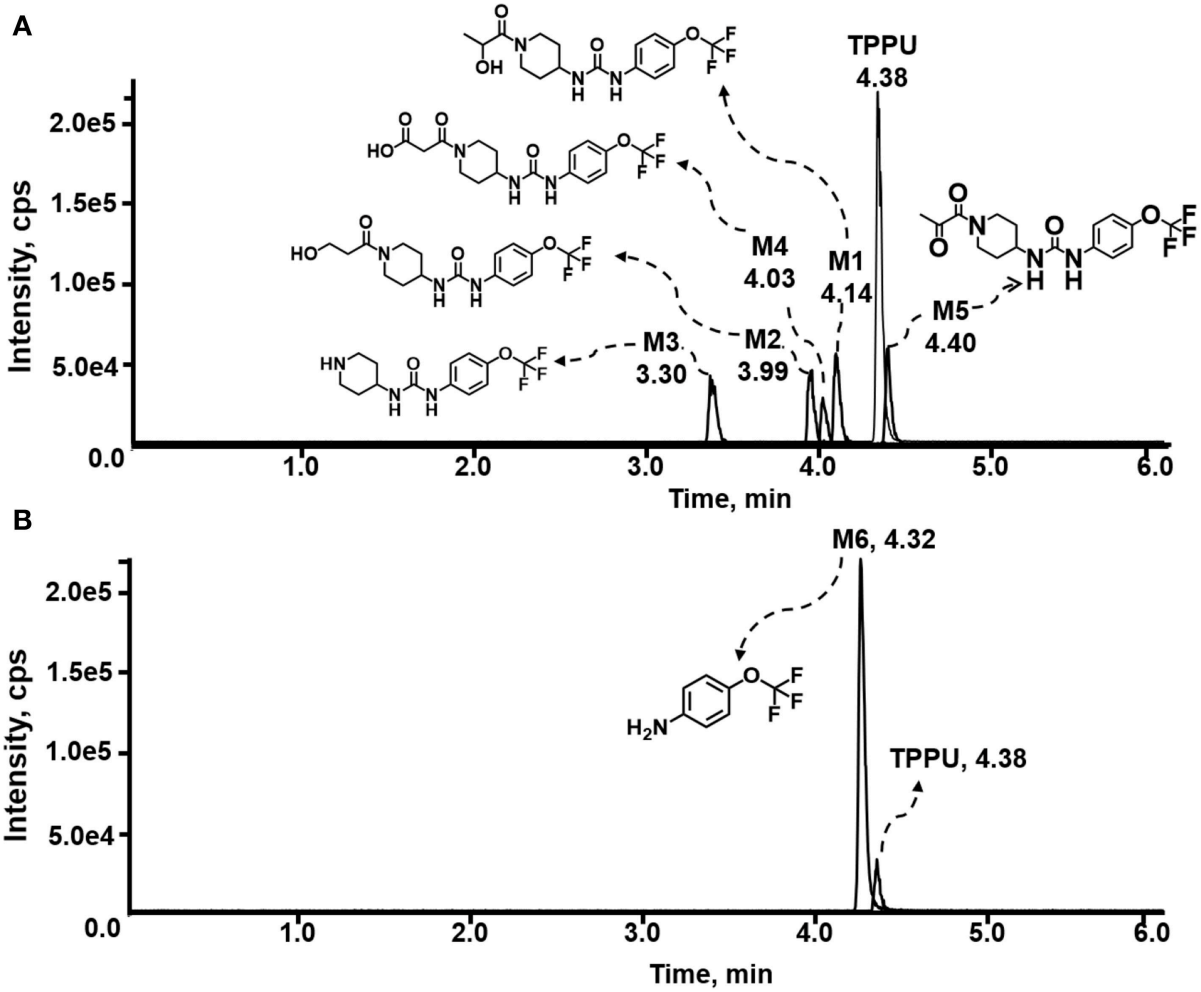
LC-MS/MS analysis of TPPU putative and synthesized metabolites. The mass spectrometric analysis was conducted under both **(A)** negative and **(B)** positive ion modes. **(A)** The chromatogram of TPPU, metabolites M1–M4, and α-keto amide (M5) metabolite. **(B)** The chromatogram of TPPU and 4-(trifluoromethoxy)aniline (M6).

A good understanding of the fragmentation pattern of TPPU in the tandem mass spectrum is critical for screening and structural identification of the TPPU metabolites. As shown in Figure 1, two major dissociation pathways were observed for TPPU in the negative ion mode tandem mass spectrometric analysis of TPPU. TPPU generated one major fragment ion at *m/z* 176 by lower-energy collision induced dissociation (CID, 22 eV), which is derived from the urea group cleavage (Figure 1A). The other minor fragment ion at *m/z* 85 appeared at higher-energy collision induced dissociation (CID, 38 eV) and is derived from the C-O bond cleavage on the trifluoromethoxyphenyl group (Figure 1B). To support the structural identification of TPPU metabolites, six putative metabolites, M1–M4, α-keto amide metabolite and 4-(trifluoromethoxy)aniline were synthesized and analyzed by LC-MS/MS (Figure 2 and Table 2). In the negative ion mode, the fragment ion at *m/z* 176 was generated by all five synthetic putative metabolites (M1–M4 and α-keto amide metabolite). Apparently, this fragment ion (*m/z* 176) is a characteristic fragment that is generated by metabolites containing the 4-(trifluoromethoxy)aniline moiety. Therefore, precursor ion scan for *m/z* 176 can be used to screen for this kind of TPPU metabolite. The method limit of detection (LOD) is 2 ng/mL. In addition, the fragment ion at *m/z* 85 (from the trifluoro-methoxy) was also generated by these synthetic standards. Presumably, this fragment ion at *m/z* 85 can be generated by metabolites resulting from oxidation and/or hydrolysis of the amide-function of TPPU regardless of the sites of metabolic conversion. Therefore, precursor ion scan for *m/z* 85 can unveil a large number of possible TPPU metabolites, such as the ones-formed from amide hydrolysis and oxidation at aliphatic groups, aromatic ring, and nitrogen center. On the basis of different ion intensity, the sensitivity of precursor ion scan for *m/z* 85 was ~5-fold lower than that of precursor ion scan for *m/z* 176. Therefore, an LC-MS/MS method with double precursor ion scans for both *m/z* 85 and *m/z* 176 was performed to screen metabolites, and an LC-MS/MS method with product ion scan was subsequently performed to support the structures of these metabolites.

**Table 2:** LC-MS/MS analysis of TPPU and synthetic putative TPPU metabolites.

**Meta. ID**	**RT (min)**	**Molecular ion (m/z)**	**Key fragments (m/z)**	**Fragmentation pattern**
TPPU	4.38	358	176, 85	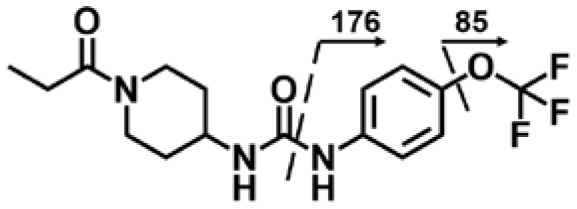
M1	4.14	374	356, 176, 85	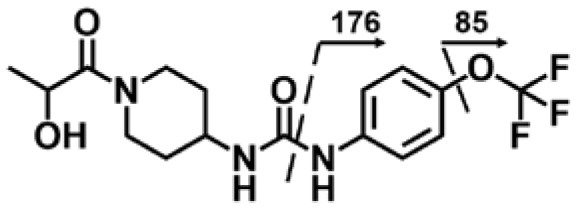
M2	3.99	374	356, 176, 85	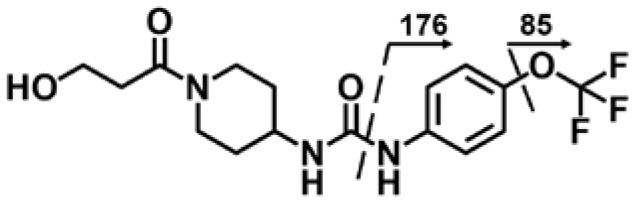
M3	3.3	302	176, 85	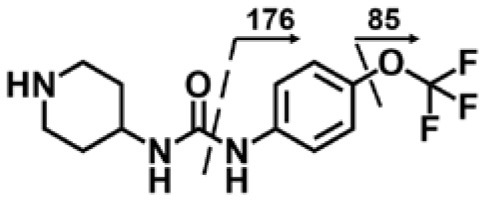
M4	4.03	388	344, 176, 85	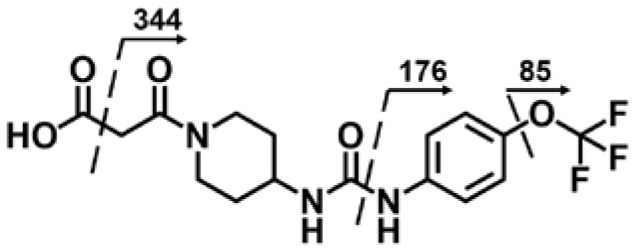
α-Keto amide (M5)	4.4	372	176, 85	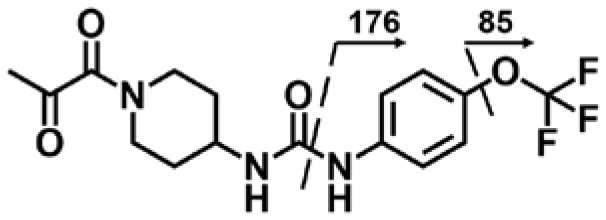
Aniline (M6)	4.32	178	93	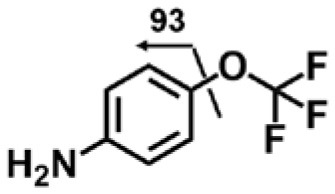

*Fragment ions of TPPU, M1–M4, and α-keto amide metabolite were obtained by product ion scan in negative-ion mode. Fragment ions of aniline was obtained by product ion scan in positive-ion mode*.

**Figure 3:**
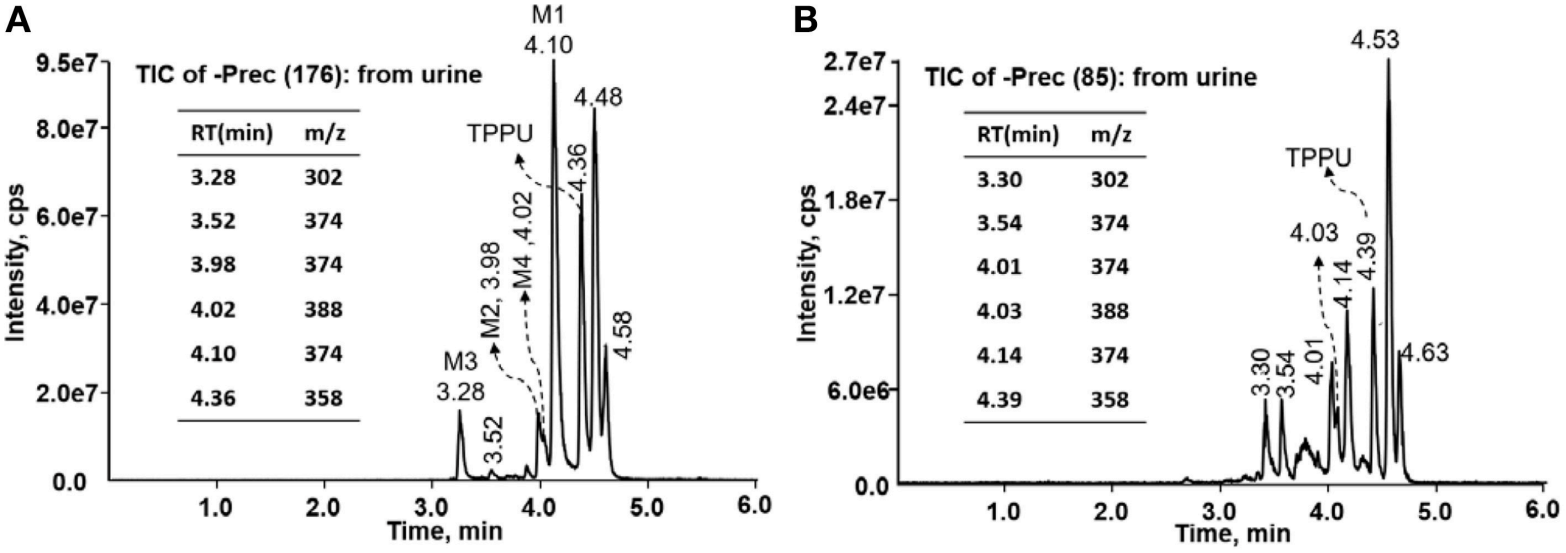
LC-MS precursor ion scan analysis of TPPU and its metabolites in rat urine extract. Metabolites in urine were extracted directly via protein precipitation by addition of acetonitrile. **(A)** Possible TPPU metabolites containing the intact 4-(trifluoromethoxy)aniline moiety in their structures were screened by precursor ion scan for *m/z* 176. **(B)** TPPU metabolites if formed without urea cleavage were screened by precursor ion scan for *m/z* 85. Insert: the retention time and corresponding *m/z* of isolated metabolites.

**Figure 4:**
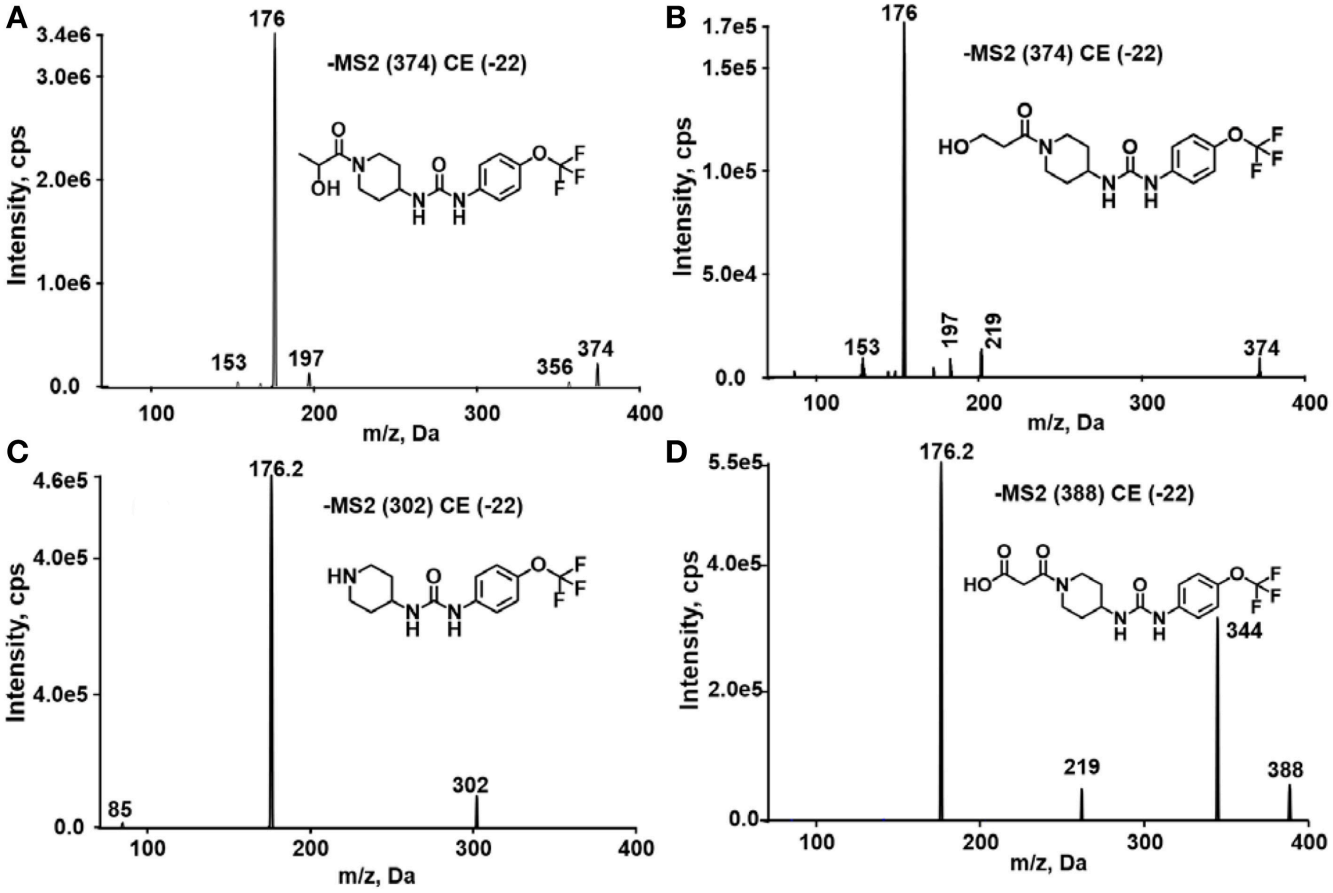
Tandem mass spectrometric analysis of TPPU metabolites eluted in Figure 3. **(A)** MS/MS analysis of ion (*m/z* 374) eluted at 4.1 min. **(B)** MS/MS analysis of ion (*m/z* 374) eluted at 3.98 min. **(C)** MS/MS analysis of ion (*m/z* 302) eluted at 3.28 min. **(D)** MS/MS analysis of ion (*m/z* 388) eluted at 4.02 min.

TPPU could be excreted via different routes, including urine, bile, sweat, saliva, lachrymation, and feces. Because of the relatively small molecular weight and high level of absorption of TPPU, we anticipated that the major routes of its excretion were via urine and feces. Therefore, to identify the major metabolite of TPPU, we investigated which metabolites were detected in urine samples collected 12 to 48 h after oral dosing. Metabolites in urine were directly analyzed by the LC-MS/MS with precursor ion scan method. As shown in Figures 3A,B, both precursor ion scan spectra for *m/z* 176 and *m/z* 85 show four peaks that were eluted earlier than TPPU, and they were M1, M2, M3, and M4, respectively. In Figure 4, TPPU eluted at 4.36 min (Figure 3A) and 4.39 min (Figure 3B) has its deprotonated ion [M–H]^−^ at *m/z* 358. Both M1 and M2 (Figures 4A,B) have deprotonated molecular ions at *m/z* 374 (358 + 16) and generate the fragment ion from loss of one molecule of H_2_O, suggesting that they are hydroxylated TPPU metabolites. M1 and M2 generate the same major fragment ion at *m/z* 176 (CID, 22 eV) derived from urea group cleavage (Figures 4A,B), indicating that hydroxylation occurred on 1-propionylpiperidinyl moiety of the TPPU molecule. By comparing the retention time of synthetic standard metabolites, M1 and M2 were elucidated to be 1-(1-(2-hydroxypropanoyl)piperidin-4-yl)-3-(4-(trifluoromethoxy)phenyl)urea (**M1**) and 1-(1-(3-hydroxypropanoyl)piperidin-4-yl)-3-(4-(trifluoromethoxy)phenyl)urea (**M2**), respectively. The metabolite at 3.54 min in the precursor ion scan spectrum of *m/z* 85 (Figure 3B) has its protonated molecular ion at *m/z* 374. In contrast, the metabolite eluted at 3.52 min in the precursor ion scan spectrum of *m/z* 176 only shows a very small peak (Figure 3A), which may indicate probable reaction on 4-(trifluoromethoxy)aniline moiety in the structure of the metabolite. Because the strong electron withdrawing capabilities of the trifluoromethoxy substitute could deactivate the aromatic ring, the metabolite at 3.54 min in Figure 3B is presumably assigned as a metabolite from hydroxylation on nitrogen atom in urea closed to phenyl moiety (compound 5 in Table 1). M3 eluted at 3.23 min (Figures 3A,B) with its protonated molecular ion at *m/z* 302 (358–56) generates major fragment ion at *m/z* 176 and minor fragment ion at *m/z* 85 in its tandem mass spectrum (Figure 4C). These data suggest that 4-(trifluoromethoxy)phenyl) moiety remained intact, and based on the molecular mass, M3 was tentatively identified as a metabolite from amide hydrolysis, and the structure elucidated by comparing the retention time, molecular ion peak and MS/MS fragmentation patterns of synthetic standard metabolite to be 1-(piperidin-4-yl)-3-(4-(trifluoromethoxy)phenyl)urea (**M3**). M4 at 4.02 min in Figure 3 has its molecular ion peak at *m/z* 388 (358 + 30) (Figure 4D). In addition to the major fragment ion at *m/z* 176, a minor fragment ion at *m/z* 344 (388–44) is observed in the tandem mass spectrum of M4 (Figure 4D). The neutral loss of 44 Da is tentatively assigned as the loss of CO_2_, suggesting that there is a carboxyl functional group in M4. By comparing the retention time, molecular ion peak and MS/MS fragmentation patterns of the corresponding synthetic standard metabolite, it was confirmed to be 3-oxo-3-(4-(3-(4-(trifluoromethoxy)phenyl)ureido)piperidin-1-yl)propanoic acid (**M4**). Based on the molecular weight shift of 14 Da between M2 (374) and M4 (388), most plausibly **M4** is one of the metabolites formed from further oxidation of the hydroxylated metabolite of TPPU (**M2**). Analysis of the urine revealed the presence of another unknown substance that eluted at 4.48 min (Figure 3A) with its molecular ion peak at *m/z* 388. This could be an unknown metabolite of TPPU. In general, drug metabolism produces more polar and water-soluble metabolites to facilitate their excretion. However, based on its relative elution time, this compound is less polar than the parent TPPU, and thus it is unlikely to be a true metabolite. Nevertheless, further studies are necessary to identify this substance.

To improve sensitivity for detection, a negative LC-MRM-MS method (Table S5) was established to identify the putative α-keto amide metabolite (compound 1 in Table 1). No peak corresponding to compound 1 was identified in urine extracts, suggesting that the putative α-keto amide metabolite if formed is lower than the detection limitation (LOD = 0.5 ng/mL).

#### Analysis of TPPU Metabolites Formed by Urea Cleavage

4-(Trifluoromethoxy)aniline is a putative metabolite that can be generated by urea cleavage. Therefore, a positive LC-MRM-MS method (Table S5) was developed for highly sensitive detection of 4-(trifluoromethoxy)aniline. However, there were no peaks corresponding to the expected aniline metabolite found from analysis of rat urine. Therefore, for the putative metabolites, 4-(trifluoromethoxy)aniline, if formed in rat, was either lower than the method detection limitation (0.1 ng/mL) or quickly conjugated to form Phase II metabolites. We further analyzed its presumed conjugates below.

### Preliminary Study of Glucuronide Conjugates and Sulfate Conjugates in Urine

Glucuronidation is a primary Phase II metabolic pathway in mammals. Based on the chemical structure, predictive Phase I metabolites by hydroxylation, hydrolysis of amide, and breakdown of urea, as well as the TPPU molecule itself, could be further metabolized to form glucuronide or sulfate conjugates, even though sulfation is usually less extensive than glucuronidation. Biliary excretion of these conjugates can either end up in feces or be recirculated and further metabolized for urinary excretion. To assess the extent of Phase II metabolism, we did a preliminary study to directly analyze possible conjugated metabolites of TPPU by LC-MS methods. However, a detailed analysis of Phase II metabolism of TPPU is beyond the scope of the study, and we will describe it separately. A neutral loss scan for 176 in both positive and negative ion modes (Table S6) was performed with rat urine to detect glucuronide conjugated metabolites of TPPU. In addition, a precursor ion scan for *m/z* 97 in negative mode (Table S7) was used to detect any possible sulfate conjugates. As a result, we were not able to identify either glucuronide or sulfate conjugates from either TPPU or its Phase I metabolites in rat urine. As reported, the related urea triclocarban (TCC) forms N- and N'-glucuronides (Schebb et al., 2012), however, we specifically searched for these possible metabolites without success. It is likely that Phase II metabolites of TPPU exist, however, we were not able to visualize them using current methods. The presumed formation of Phase II conjugated metabolites in rat feces and urine will be further characterized by enzymatic hydrolysis and acid hydrolysis analysis with separate analysis using authentic standards.

### *In vitro* Metabolism of TPPU: Species Comparisons

**Figure 5:**
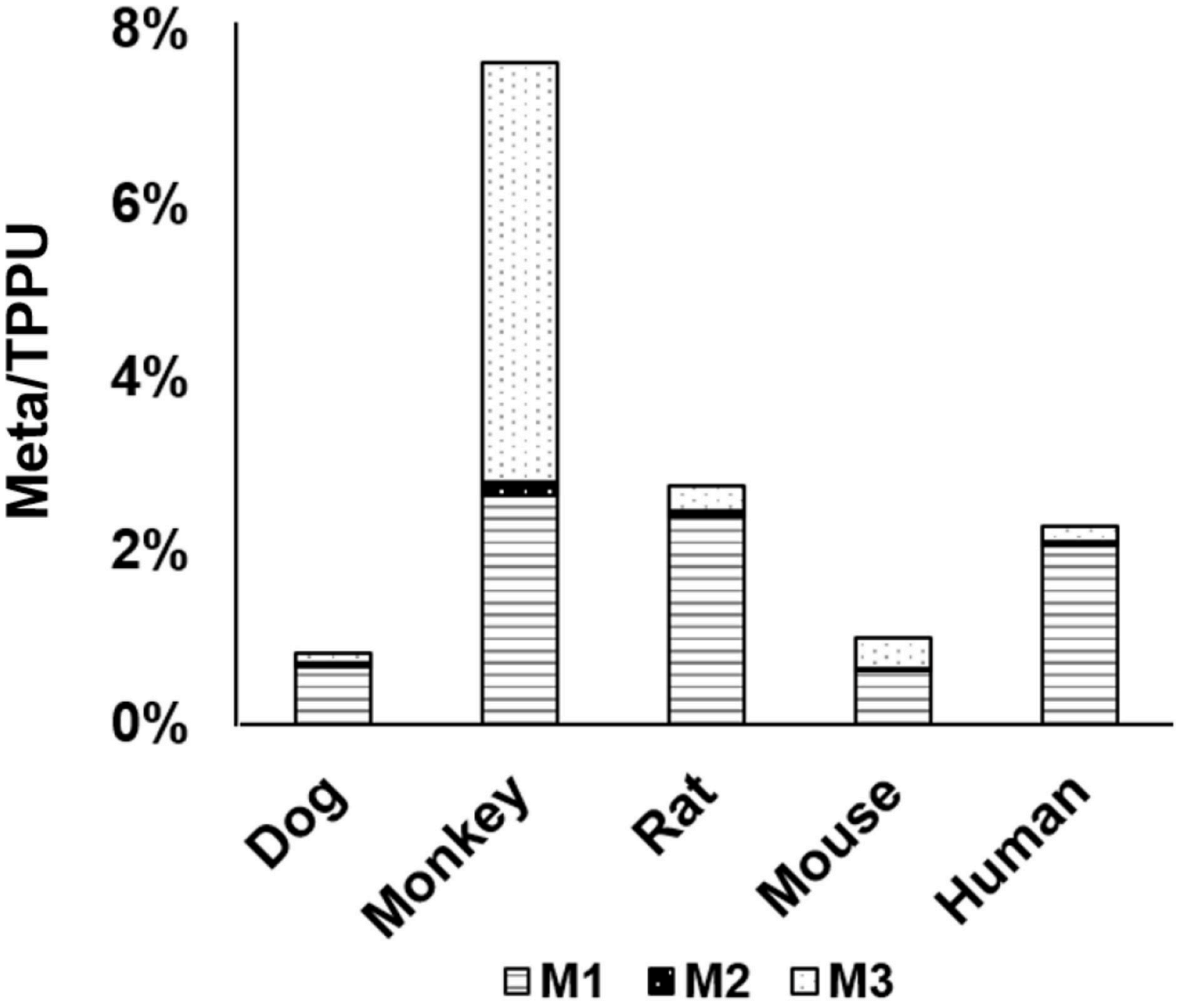
Relative abundance of metabolites M1–M3 formed in liver S9 fractions from five different species: human, monkey, dog, rat, and mouse.

After identifying metabolites in rat urine, we determined the interspecies difference in the metabolism of TPPU by *in vitro* incubation with liver S9 fractions. The liver is the primary location for metabolism that drugs are likely to encounter; and therefore, it was informative to test the metabolism of TPPU *in vitro* by using liver S9 fractions. TPPU was incubated using five liver S9 fractions obtained from different species, namely rat, mouse, dog, monkey, and human. TPPU metabolites M1, M2, and M3 were found in all species tested (Figure 5). However, M4 was not detected in the S9 fractions from any of the species. *In vitro* metabolism of TPPU varied significantly among these five different species. The S9 from monkey liver generated the greatest amount of metabolite M3. This was presumably from amide hydrolysis since it was formed in similar amounts with and without a NADPH generating system (Figure S1). The S9 fractions from rat, mouse, dog, and human generated TPPU metabolites primarily from hydroxylation, and the TPPU metabolites followed the same M1>M3>M2 order. Similar quantities of TPPU metabolites were generated by rat and human liver S9 fractions, indicating high degree of similarity of TPPU metabolism between these two species. Based on the disappearance of TPPU, the order of liver S9 activities for TPPU metabolism appeared to be monkey > rat ≈ human > mouse > dog.

### *In vivo* Metabolism of TPPU

**Figure 6:**
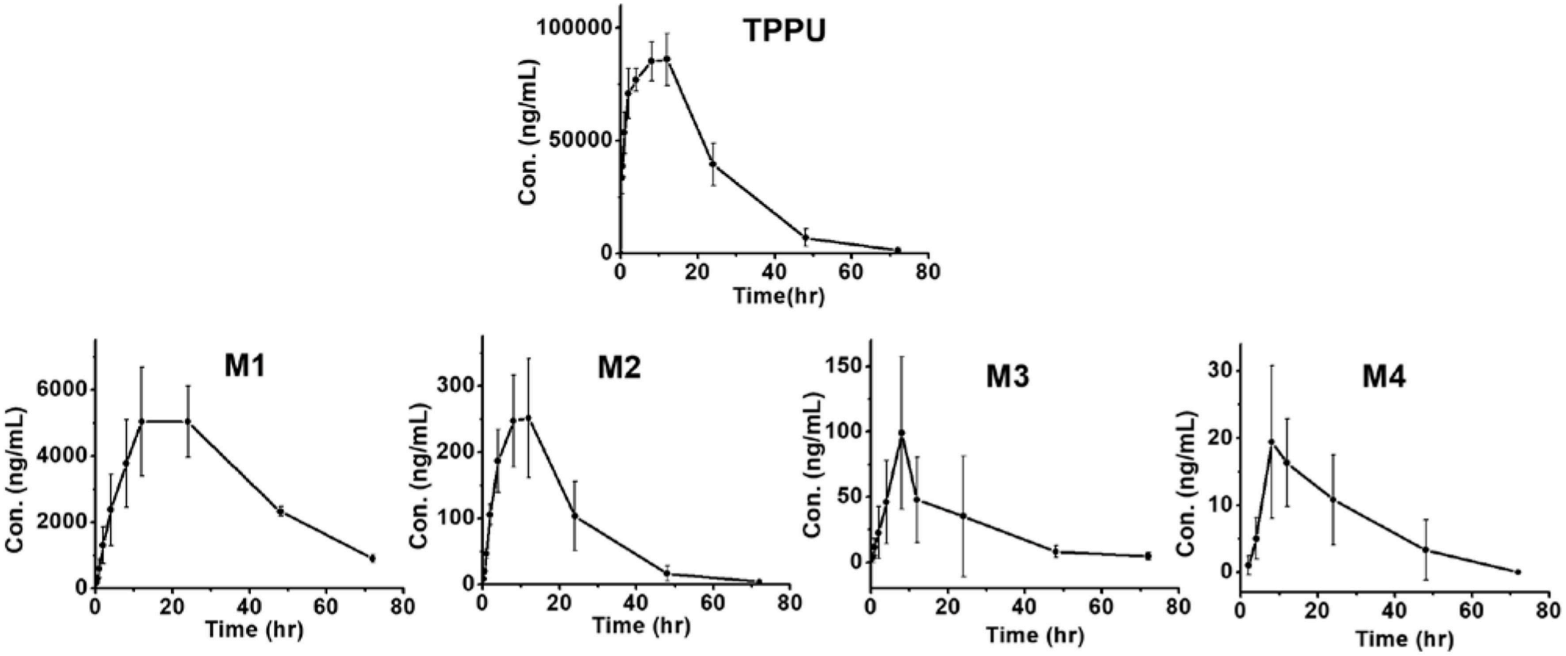
Time course of blood TPPU and its metabolites levels in rat blood following a single oral dose of 10 mg/kg (*n* = 4).

**Figure 7:**
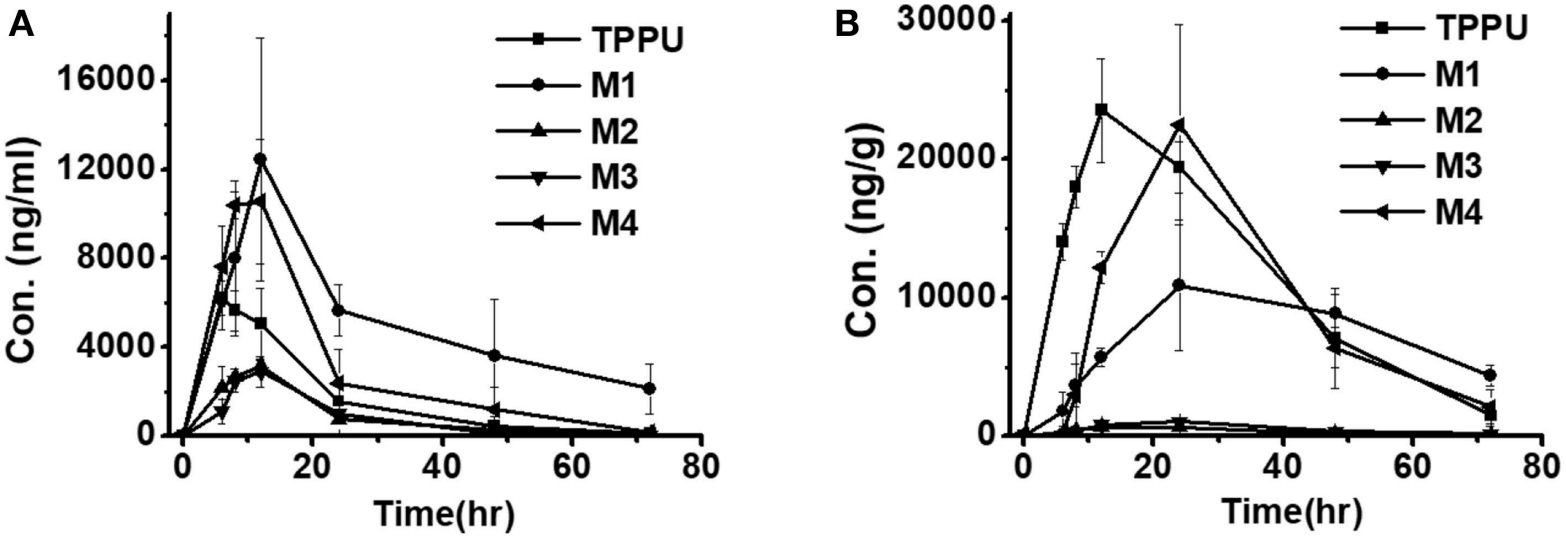
Time course of TPPU and its metabolite levels in rat urine **(A)** and feces **(B)** following a single oral dose of 10 mg/kg (*n* = 3).

Blood concentrations over time of TPPU and its metabolites were further investigated in rats. Consistent with the data obtained in the *in vitro* rat liver S9 studies, metabolite M1 derived from TPPU hydroxylation was the major circulating metabolite in rat blood (Figure 6). In contrast, M2 instead of M3 was found to be the second major metabolite in blood, and the oxidative metabolite (M4) was found both in urine and blood, demonstrating that the metabolite M2 was further oxidized *in vivo*. Other presumable oxidative metabolites, α-keto amide metabolite and 4-(trifluoromethoxy)aniline derived from urea breakdown were not detectable. In addition, no glucuronide and sulfate conjugated metabolites in urine were identified by LC-MS/MS analysis. TPPU was metabolized via phase I reactions, including hydroxylation (M1 and M2), amide hydrolysis (M3), and oxidation (M4). These results suggest that TPPU is metabolized predominantly via oxidation and secondary via amide hydrolysis. Furthermore, three of these four metabolites rapidly appear in the circulation because they were present 0.25 h in blood after dosage. On the other hand, over 90% the TPPU nucleus in the blood was present as the parent compound (Table 3) indicating either high stability or rapid conjugation and excretion of the more polar metabolites. Then, concentrations of TPPU metabolites as well as unchanged TPPU in both urine and feces were further determined. A large amount of TPPU metabolites (3.4 × 10^4^ ng/mL at C_*max*_ of 12 h) and unchanged TPPU (5.7 × 10^3^ ng/mL at C_*max*_ of 8 h) were found in urine (Table S8), while even more metabolites (5.5 × 10^4^ ng/mL at C_*max*_ of 24 h) and unchanged TPPU (2.4 × 10^4^ ng/mL at C_*max*_ of 12 h) were found in feces (Table S9). M1, M4 and unchanged TPPU were found to be major substances in urine and feces, shown in Figure 7. M1 was the major metabolite in urine with its *C*_*max*_ of 1.2 × 10^4^ ng/mL at 12 h (Figure 7A and Table S8), and unchanged TPPU and M4 were determined to be the major substances in feces with their *C*_*max*_ of 2.4 × 10^4^ ng/mL at 12 h and 2.2 × 10^4^ ng/mL at 24 h (Figure 7B and Table S9), respectively. That more M4 than M2 found in both urine and feces further indicates M2 was probably further oxidized to M4 to facilitate its excretion. Based on the comparison of total substances in urine and feces and TPPU in blood, it seems TPPU was majorly eliminated as Phase I metabolites and parent drug via urine and feces. In general, ω- and ω-1 aliphatic hydroxylation is anticipated to be far more rapid than aromatic hydroxylation, because the strong electron withdrawing by the trifluoromethoxy substitute deactivated the aromatic ring.

**Table 3:** TPPU and its identified Metabolites: chemical formula, structures, inhibitory potency toward human she, and non-compartmental pharmacokinetic parameters after oral gavage at a 10 mg/kg in rats (*n* = *4*).

**Meta. ID**	**Formula**	**HsEH IC50^**a**^ (nM)**	AUC^**b**^ (nM^*^h)	***C_**max**_*^**c**^ (nM)**	***T_**1/2**_*^**d**^ (h)**
TPPU	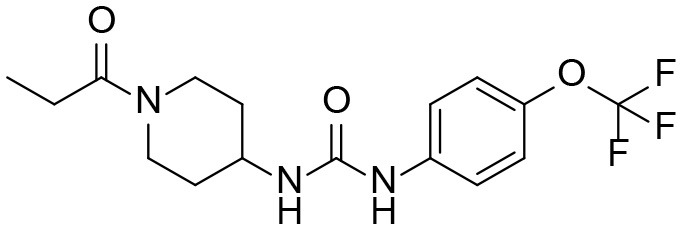	1.1 ± 0.1	6.0 ± 0.9 10^6^	2.0 ± 0.3 10^5^	9.0 ± 1.8
	1-(1-propionylpiperidin-4-yl)-3-(4-(trifluoromethoxy)phenyl)urea				
M1	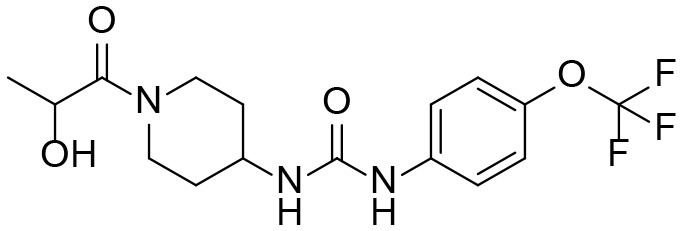	3 ± 0.5	6.0 ± 1.0 10^5^	1.0 ± 0.4 10^4^	20 ± 3
	1-(1-(2-hydroxypropanoyl) piperidin-4-yl)-3-(4-(trifluoromethoxy)phenyl)urea				
M2	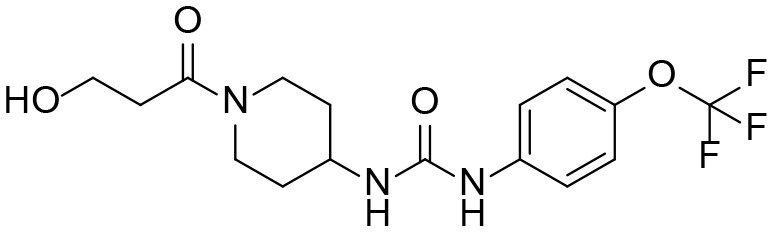	16 ± 2	2.0 ± 0.6 10^4^	7.0 ± 2.0 10^2^	9.7 ± 0.8
	1-(1-(3-hydroxypropanoyl) piperidin-4-yl)-3-(4-(trifluoromethoxy)phenyl)urea				
M3	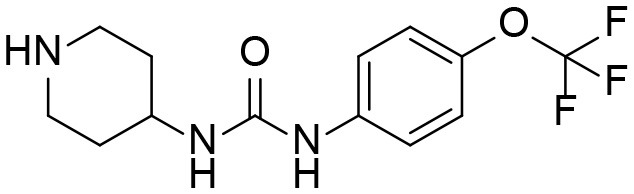	83 ± 9	6.0 ± 4.0 10^3^	3.0 ± 2.0 10^2^	14 ± 2.7
	1-(piperidin-4-yl)-3-(4-(trifluoromethoxy)phenyl)urea				
M4	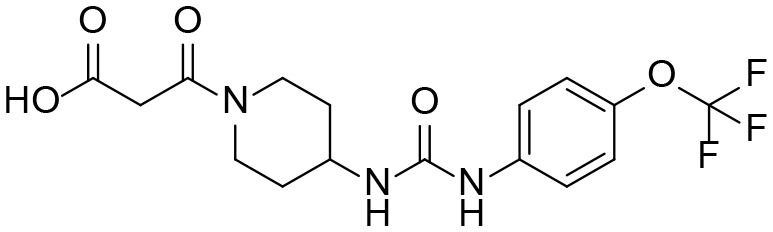	158 ± 10	1.0 ± 0.4 10^3^	53 ± 28	17 ± 4.7
	3-oxo-3-(4-(3-(4-(trifluoromethoxy)phenyl)ureido) piperidin-1-yl)propanoic acid				

a *IC50 values were determined by CMNPC fluorescent assay*.

b *Area under the concentration (Time_0−72h_)*.

c *Maximum blood concentration*.

d *half-life*.

### The Potencies of TPPU and Its Metabolites

Because all four metabolites retained the central pharmacophore of *N,N'*- disubstituted urea in the structure, they maintained sEH inhibition and a portion of the drug effect of TPPU may be associated with these metabolites. In Table 3, these four major TPPU metabolites still have sEH inhibitory activity, although their inhibitory potencies are weaker than that of TPPU (IC_50_ = 1 nM). Among all TPPU metabolites analyzed, the hydroxylated metabolite M1 has the highest inhibitory potency (IC_50_ = 3 nM) toward human sEH, which is ~5-fold more potent than that of hydroxylated metabolite M2 (IC_50_ = 16.7 nM). This indicates that the hydroxylation position significantly influences the sEH inhibitory activity. M3 derived from amide hydrolysis has 80-fold lower potency (IC_50_ = 83.5 nM) than that of TPPU (1 nM), indicating that having the free amine of the piperidinyl group significantly reduces sEH inhibitory potency. M4 possessed the lowest potency (IC_50_ = 158 nM) among these four metabolites. This demonstrates that having the free carboxyl group significantly reduces sEH inhibitory potency. The comparison of sEH inhibitory activities of TPPU and its metabolites is helpful to gain knowledge of relationship between chemical structure and sEH inhibitory potency.

## Discussion

The sEH inhibitors were designed to inhibit the transition state of the enzyme from metabolizing the epoxide to product diol. Early chemical efforts (Rose et al., 2010) and computational studies (Waltenberger et al., 2016) found that most potent inhibitors have urea or carbamate pharmacophores. Two of the compounds, AR9281 and GSK2256294, published results from Phase I clinical trials with no significant safety concerns (Watanabe et al., 2003; Liu et al., 2015), while the third, EC5026 from EicOsis, is being prepared for human phase I safety studies. There is increasing interests in the development of sEH inhibitors for the treatment of a number of diseases. However, the metabolism of few sEHI has been studied so far, which is an important aspect of the drug development of sEHI. Previously, 1-cyclohexyl-3-dodecyl-urea (CDU) and 1-adamantan-1-yl-3-(5-(2-(2-ethoxyethoxy)ethoxy)pentyl)urea (AEPU) was studied for its Phase I metabolism and a hydroxylated metabolite was found to be metabolically more stable than its parent and retain some potency on the sEH target (Chen et al., 2012; Lazaar et al., 2016). Published clinical trial results with AR9281 and GSK2256294 indicate that oxidative metabolism is the primary route of their metabolism in humans, and saturation at high doses reported with AR9281 results in non-linear kinetics. Although the metabolites were not reported for either compound, the formation of oxidative metabolism without presence of glucuronide metabolites was listed as the major route of elimination for both compounds, similar to the metabolites we identified for TPPU. Because the metabolites retained the pharmacophore, it is important to understand the potency of these metabolites in order to identify an accurate therapeutic index and safety profile. More information of the relationship between chemical structure and sEH inhibitory potency of 1-Aryl-3-(1-acylpiperidin-4-yl) urea inhibitors has been reported by Rose et al. (2010).

Preclinical metabolism is often overlooked in driving small molecules to human clinical development; however, better characterization of metabolism early in development may help avoid unexpected consequences in clinical trials, thus preventing expensive failures late in development. The challenge to this approach is that early metabolism studies are unique to each compound and require a high level of technical expertise. This study reports methods and results for identifying unknown Phase I metabolites that can be applied to other small molecules. Although the metabolites are specific to this compound, they can be used to assess metabolism of other similar compounds. More importantly, these methods will identify potential major metabolites; and by assessing in different species, it reduces the risk of finding major metabolites in human trials, when toxicity profiles may not have been established in IND-enabling toxicity studies. Although no major metabolites were identified for TPPU in blood, understanding how metabolism affects efficacy is important for designing safe clinical trials, especially when low concentrations of a more potent metabolite could influence safety and efficacy. For example, we found that the hydroxylation position significantly influences sEH inhibitory activity. The major metabolite M1 still has sub nanomolar potency and an *in vivo* t_1/2_ that is much longer than TPPU. Therefore, even low concentrations of this metabolite may play a significant role in the efficacious profile for this drug. Similar effects of different hydroxylation positions on sEH potency have been also observed for N-adamantyl substituted urea-based sEH inhibitor (Lazaar et al., 2016). In this manuscript, we studied the relationship between different metabolites and their sEH inhibitory potency. However, further studies should be conducted to determine if the relationship is applicable for all sEH inhibitors.

In the present study, LC-MS/MS methods were employed to investigate *in vitro* and *in vivo* metabolism of TPPU. Based on the unique fragmentation pattern of TPPU and synthetic standard metabolites in tandem mass spectrum, we employed a LC-MS/MS method with double precursor ion scans for *m/z* 85 (CID, 38 eV) and *m/z* 176 (CID, 22 eV) to identify TPPU metabolites. The LC-MS/MS method with double precursor ion scans has some distinct advantages. Most importantly, the ion chromatograms obtained by precursor ion scan showed clear peaks corresponding to these compound metabolites (Figure 3). Using this highly sensitive LC-MS/MS method, we identified four TPPU metabolites *in vivo*, including M1 and M2 formed from ω and ω-1 hydroxylation of TPPU, M3 from amide hydrolysis of TPPU on 1-propionylpiperdine residue, and M4 from further oxidation of terminal hydroxylated metabolite M2. Two putative metabolites, including 4-(trifluoromethoxy)aniline derived from urea cleavage and the α-keto amide metabolite, were not identified in urine even by highly sensitive LC-MRM-MS methods. In urine, neither glucuronide or sulfate conjugates formed from TPPU and its Phase I metabolites were detected by neutral loss scanning for 176 Da and precursor ion scanning for *m/z* 97 based LC-MS/MS methods. The other predicted metabolites of TPPU in Table 1, if formed, were present at a concentration lower than 2 ng/mL (LOD). Their presence was not confirmed due to their very low concentrations and lack of synthetic standards.

**Figure 8:**
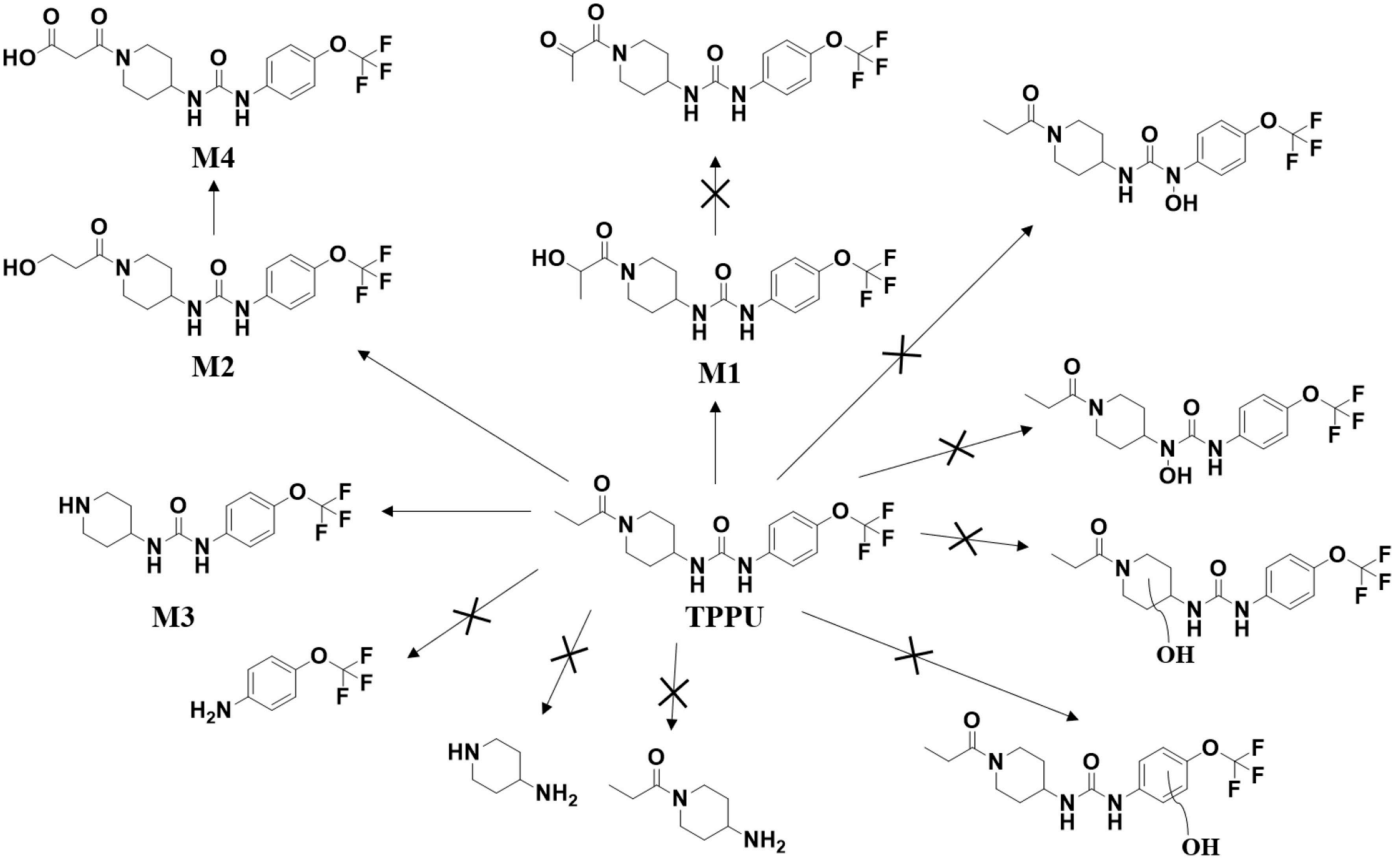
Major routes of Phase I metabolism of the sEHI TPPU detected in rat by LC-MS/MS. The pathways not detected are marked by X.

Both *in vitro* and *in vivo* data showed that the metabolism of TPPU mainly occurred on 1-propionylpiperidinyl moiety of the molecule but not on 4-(trifluoromethoxy)phenyl moiety. Consistent with the result from previous report (Ostermann et al., 2015), TPPU shows good metabolic stability when incubated with liver S9 fractions, as over 90% of TPPU remained after 2 h of incubation. M1, M2, and M3 were observed when TPPU was incubated in liver S9 fractions in presence of a NADPH generating solution. In the absence of a NADPH generation system, M1 and M2 failed to be detected whereas M3 could still be produced in liver S9 fractions (Figure S1). This suggests that hydroxylation of TPPU is mainly through CYP enzymes, while amide hydrolysis of TPPU is probably dependent on amidases or other hydrolytic enzymes. *In vivo* data showed that TPPU was predominately metabolized via three metabolic pathways, hydroxylation, amide hydrolysis and oxidation, with hydroxylation being the dominant process (Figure 8). On the basis of the *t*_1/2_ values in Table 3, M2 was probably excreted more rapidly than M1 due to its relatively higher polarity, which is consistent with their elution order in chromatography (Figure 2). In rat blood, <10% of TPPU is present as its metabolites. This observation suggests that these more polar metabolites are rapidly conjugated and excreted after formation. A large amount of TPPU metabolites were found both in urine and feces. In addition, TPPU itself can be also detected in both urine and feces samples after 8 h. Therefore, the excretion of TPPU was determined to be majorly via Phase I metabolism and parent drug in urine and feces. Overall the data suggest that the pathways for TPPU excretion include primarily phase I metabolism and self-elimination. The good absorption and relatively long half-life in the blood likely contribute to its high potency and target occupancy of TPPU in multiple disease states (Kodani and Hammock, 2015). M1 with its IC_50_ at 3 nM and relatively longer half-life (~20 h) could contribute to *in vivo* inhibition. The much higher IC_50_ values of M2 and M3 and relatively short half-life than M1 make them unlikely to contribute significantly to the pharmacological efficacy of TPPU *in vivo*.

In conclusion, we demonstrated species differences of TPPU metabolism using liver S9 fractions and identified TPPU metabolites in both rat blood and urine. TPPU underwent phase I metabolism, mostly through hydroxylation (M1 and M2), followed by amide hydrolysis (M3) and one of the hydroxylated metabolites (M2) was subject to further oxidation to form M4. The failure to find metabolites of TPPU hydroxylated on the aromatic ring or the piperidine ring suggests that these are likely minor pathways of metabolism. It is also possible that these putative metabolites could have been formed and rapidly metabolized to other Phase II metabolites that is not detected. As has been reported for other sEHI, we failed to find possible metabolites resulting from urea cleavage. The 1-propionylpiperidinyl moiety on TPPU is the most metabolically vulnerable part in the molecule. These insights inform the predicted metabolism of other sEH inhibitors containing the acyl-piperidinyl group and a urea central pharmacophore. Therefore, it provides useful information for the optimization of future sEH inhibitors for translation to the clinical environment.

## Ethics Statement

All animal studies were approved by the UC Davis IACUC committee.

## Author Contributions

DW, JY, CBM, and BDH designed research. DW, BB, KW, CM, JS, and RB performed research. SH contributed TPPU and 4-(trifluoromethoxy)aniline. DW, JY, CBM, BB, KW, SH, and BDH analyzed data and edited manuscript.

### Conflict of Interest Statement

BDH and CBM are co-founders. JY and KW are employees of EicOsis, a company advancing sEH inhibitors as potential therapeutics. The remaining authors declare that the research was conducted in the absence of any commercial or financial relationships that could be construed as a potential conflict of interest.
