# Supplementary material for: In vitro and in vivo Metabolism of a Potent Inhibitor of Soluble Epoxide Hydrolase, 1-(1-Propionylpiperidin-4-yl)-3-(4-(trifluoromethoxy)phenyl)urea
Source: Front Pharmacol. 2019 May 8;10:464. doi: 10.3389/fphar.2019.00464 (PMC6520522; doi:10.3389/fphar.2019.00464)
Supplement: Supplementary file 1 [file Data_Sheet_1.docx]

**Supplementary Material**

*In Vitro* and *In Vivo* Metabolism of a Potent Inhibitor of Soluble Epoxide Hydrolase, 1-(1-Propionylpiperidin-4-yl)-3-(4-(trifluoromethoxy)phenyl)urea

**Debin Wan,^1^ Jun Yang,^1^ Cindy B. McReynolds,^1^ Bogdan Barnych,^1^ Karen M. Wagner,^1^ Christophe Morisseau,^1^** [**Sung Hee Hwang**](https://plus.google.com/u/0/116368653422818293624?prsrc=4)**,^1^ Jia Sun,^1,2^ René Blöcher,^1^ and Bruce D. Hammock^1,^***

^1^Department of Entomology and Nematology and University of California Davis Comprehensive Cancer Center, University of California, Davis, Davis, CA 95616

^2^State Forestry Administration Key Open Laboratory, International Center for Bamboo and Rattan, Beijing 100102, China

*** Correspondence:**Bruce D. Hammock
[bdhammock@ucdavis.edu](mailto:bdhammock@ucdavis.edu)

**Table S-1. LC gradient for *in vivo* study**

| Time (min) | B (%) |
| --- | --- |
| 0 | 20 |
| 1 | 20 |
| 4 | 80 |
| 5 | 90 |
| 5.1 | 20 |
| 6 | 20 |

**Table S-2. LC gradient for *in vitro* study**

| Time (min) | B (%) |
| --- | --- |
| 0 | 10 |
| 3 | 10 |
| 3.5 | 35 |
| 6 | 60 |
| 7 | 80 |
| 7.5 | 98 |
| 8 | 98 |
| 8.1 | 10 |
| 9 | 10 |
| Time Events Enabled | 0 min, flow state sets up to waste;  3 min, flow state changes to mass spectrometer |

**Table S-3. Mass Spectrometric Source Parameters**

| Acquisition Parameters | ESI, negative | ESI, positive |
| --- | --- | --- |
| CUR: | 30 | 30 |
| TEM: | 600 | 600 |
| GS1: | 50 | 50 |
| GS2: | 60 | 60 |
| iHe: | ON | ON |
| CAD: | Medium | Medium |
| IS: | -4500 | 5500 |
| EP: | -10 | 10 |

**Table S-4. LC-PIS-MS parameters for screening Phase I metabolites of TPPU**

| Precursor of | Start (Da) | Stop (Da) | Step (Da) | DP (V) | CE (V) | CXP (V) |
| --- | --- | --- | --- | --- | --- | --- |
| 176 | 300 | 600 | 0.1 | -100 | -22 | -9 |
| 85 | 300 | 600 | 0.1 | -100 | -35 | -4 |

**Table S-5. LC-MRM-MS parameters for synthetic putative metabolites of TPPU**

| ID | Q1 | Q3 | Dwell (msec) | DP (V) | CE (V) | CXP (V) |
| --- | --- | --- | --- | --- | --- | --- |
| TPPU | 358.2 | 176.1 | 30 | -100 | -20 | -9 |
| CUDA | 339.3 | 214.2 | 30 | -65 | -32 | -4 |
| TAPU | 327.9 | 159.8 | 30 | -100 | -24 | -11 |
| M1 | 374.3 | 176.1 | 30 | -105 | -22 | -9 |
| M2 | 374.3 | 176.1 | 30 | -105 | -22 | -9 |
| M3 | 302.2 | 176.1 | 30 | -90 | -20 | -7 |
| M4 | 388.2 | 176.1 | 30 | -100 | -22 | -8 |
| M5 | 372.2 | 176.1 | 30 | -105 | -22 | -8 |
| M6 | 178.1 | 93.0 | 30 | 66 | 35 | 6 |

**Table S-6. LC-NLS-MS parameters for screening glucuronide conjugated metabolites of TPPU**

| Neutral loss of | Start (Da) | Stop (Da) | Step (Da) | DP (V) | CE (V) | CXP (V) |
| --- | --- | --- | --- | --- | --- | --- |
| 176 | 250 | 1000 | 0.1 | -110 | -20 | -9 |
| 176 | 250 | 1000 | 0.1 | 105 | 20 | 8 |

**Table S-7. LC-PIS-MS parameters for screening sulfate conjugated metabolites of TPPU**

| Precursor of | Start (Da) | Stop (Da) | Step (Da) | DP (V) | CE (V) | CXP (V) |
| --- | --- | --- | --- | --- | --- | --- |
| 97 | 200 | 1000 | 0.1 | -105 | -20 | -8 |

**Table S8. Prototype TPPU and its metabolites in urine at different time pointes. Concentrations are shown as ng/mL.**

| Time | TPPU | M1 | M2 | M3 | M4 | Sum of M1-M4 |
| --- | --- | --- | --- | --- | --- | --- |
| 8h | 5.7 ± 0.9 10^3^ | 8.0 ± 3.0 10^3^ | 2.7 ± 0.3 10^3^ | 2.5 ± 0.5 10^3^ | 1.0 ± 0.1 10^4^ | 2.9 ± 0.5 10^4^ |
| 12h | 5.1 ± 1.6 10^3^ | 1.2 ± 0.5 10^4^ | 3.2 ± 0.3 10^3^ | 2.9 ± 0.7 10^3^ | 1.1 ± 0.2 10^4^ | 3.4 ± 1.1 10^4^ |
| 24h | 1.6 ± 0.8 10^3^ | 5.6 ± 1.0 10^3^ | 7.6 ± 6.4 10^2^ | 1.0 ± 0.4 10^3^ | 2.4 ± 1.5 10^3^ | 1.1 ± 0.4 10^4^ |
| 48h | 4.6 ± 4.0 10^2^ | 3.6 ± 2.0 10^3^ | 3.0 ± 2.8 10^2^ | 1.5 ± 0.8 10^2^ | 1.2 ± 0.9 10^3^ | 5.7 ± 4.0 10^3^ |
| 72h | 1.1 ± 0.4 10^2^ | 2.1 ± 1.0 10^3^ | 61 ± 60 | 44 ± 21 | 1.7 ± 1.4 10^2^ | 2.5 ± 1.0 10^3^ |

**Table S9. Prototype TPPU and its metabolites in feces at different time pointes. Concentrations are shown as ng/mL.**

| Time | TPPU | M1 | M2 | M3 | M4 | Sum of M1-M4 |
| --- | --- | --- | --- | --- | --- | --- |
| 8h | 1.8 ± 0.1 10^4^ | 3.7 ± 1.6 10^3^ | 4.3 ± 1.9 10^2^ | 3.6 ± 3.0 10^2^ | 3.0 ± 3.0 10^3^ | 2.5 ± 0.6 10^4^ |
| 12h | 2.4 ± 0.4 10^4^ | 5.7 ± 0.6 10^3^ | 6.8 ± 1.4 10^2^ | 8.3 ± 0.5 10^2^ | 1.2 ± 0.1 10^4^ | 4.3 ± 0.5 10^4^ |
| 24h | 1.9 ± 0.2 10^4^ | 1.1 ± 0.5 10^4^ | 6.7 ± 2.2 10^2^ | 1.1 ± 0.1 10^3^ | 2.2 ± 0.7 10^4^ | 5.5 ± 1.4 10^4^ |
| 48h | 7.0 ± 3.6 10^3^ | 8.8 ± 1.4 10^3^ | 1.9 ± 1.2 10^2^ | 3.4 ± 1.3 10^2^ | 6.4 ± 1.5 10^3^ | 2.3 ± 0.6 10^4^ |
| 72h | 1.5 ± 0.8 10^3^ | 4.4 ± 0.7 10^3^ | 43 ± 31 | 1.7 ± 1.1 10^3^ | 2.1 ± 1.2 10^3^ | 8.0 ± 3.0 10^3^ |


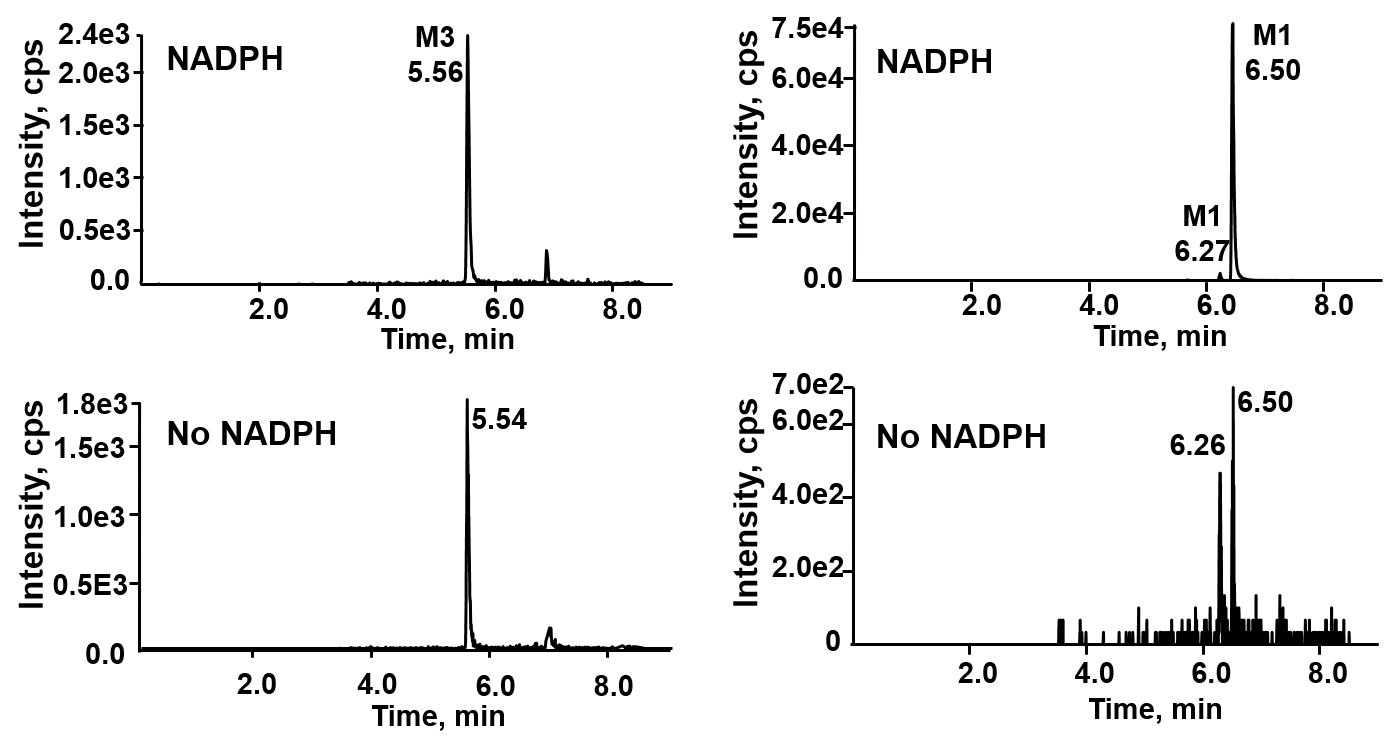


**Figure S1. LC-MS/MS analysis of TPPU metabolites in human liver S9 fractions with and without NADPH.**
